# The impact of 14-day head-down bed rest with or without an exercise countermeasure on standing balance control: a randomized controlled trial

**DOI:** 10.1038/s41526-026-00624-x

**Published:** 2026-06-26

**Authors:** Nok-Yeung Law, Karima Ahmed Yahia, Joseph Gill-Lussier, Ali Falaki, Helga Tonkov, Faezeh Abbariki, Andréa Faust, José A. Morais, Guy Hajj-Boutros, Dorothy Barthélemy

**Affiliations:** 1 Institut Universitaire de Réadaptation en Déficience Physique de Montréal, site of Center for Interdisciplinary Research in Rehabilitation of Greater Montreal (CRIR), CIUSSS du Centre-Sud-de-l’Ile-de, Montréal, QC Canada; 2 School of Rehabilitation, Faculty of Medicine, Université de Montréal, Montreal, QC Canada; 3 Research Institute of McGill University Health Centre, McGill University, Montréal, QC Canada; 4 Division of Geriatric Medicine, McGill University Health Centre, McGill University, Montréal, QC Canada

**Keywords:** Health care, Medical research, Neurology, Neuroscience, Physiology

## Abstract

Exposure to space environment disrupts the sensorimotor system due to adaptation to microgravity, leading to spatial disorientation, impaired coordination, and reduced postural control upon return to Earth. To simulate these effects, a six‑degree head‑down bed rest (HDBR) protocol was used. This randomized controlled trial examined whether exercise could mitigate declines in standing balance control following 14 days of HDBR. Twenty-two participants were assigned to either an exercise group, performing daily high-intensity interval training combined with resistance and aerobic exercise, or a non-exercising control group. Balance was assessed using the sensory organization test (SOT) and head-shake SOT, with outcomes including equilibrium score (ES), strategy analysis (SA), and vestibular-related measures (e.g., SOT-2M, SOT-5M). No between-group differences were observed in either ES or SA. However, when data were pooled, ES during SOT-2M was significantly decreased (*p* < 0.001), indicating greater instability under eyes-closed, head-shake conditions. Higher baseline ES during SOT-2M was associated with smaller declines post-HDBR, particularly in the exercise group. No effects of sex or age (55–65 years) were found. These findings suggest that exercise may not prevent balance declines after short-term HDBR and that baseline balance capacity may be associated with variability in individual responses. Clinical Trial Registration: NCT04964999 (2021-07-16).

## Introduction

Gravity serves as a crucial signal for the sensorimotor system, facilitating the perception of self-orientation in space and aiding in the adjustment of movements and posture^1^. During space missions, astronauts navigate a gravity-free environment. Over time, the sensorimotor system adapts to this unique setting^2^, leading to a modulation in the integration of sensory inputs and motor commands to optimize performance in microgravity^2^. Upon returning to Earth, these adaptations can become maladaptive, resulting in disrupted coordination of movements and posture^2^. Nearly 70% of astronauts encounter alterations in movement control and balance during and post-spaceflight^1^.

As space agencies gear up for extended-duration missions, understanding these balance control alterations induced by microgravity becomes paramount. Head-down bed rest (HDBR) is a widely used experimental model to simulate the effects of microgravity, as directly studying microgravity on astronauts is challenging. The six-degree head-down tilt mimics the redistribution of blood volume towards the upper body, similar to what occurs in space, while reducing gravitational resistance, which affects the sensory stimuli crucial for determining body position and orientation relative to gravity^3–5^. This makes HDBR an effective method for studying the deconditioning effects of microgravity and testing potential countermeasures.

In parallel, accumulating knowledge on sensorimotor adaptation has informed the development of evidence‑based countermeasures designed to facilitate astronauts’ transitions between gravitational environments and mitigate the detrimental effects associated with microgravity exposure^6^. Physical training methods such as aerobic exercise, resistance training, and high-intensity interval training (HIIT) have emerged as potential strategies, primarily targeted cardiovascular health and minimized muscle mass decline in the lower extremities^7–9^.

While exercise countermeasures in operational spaceflight have demonstrated only partial effectiveness in preventing postflight sensorimotor impairments, their specific effects on postural control and sensory integration under controlled unloading conditions remain incompletely understood. An HDBR model provides a controlled environment in which these effects can be isolated from the operational constraints and confounding factors inherent to spaceflight. This allows for a more detailed examination of postural responses to exercise-based countermeasures, as well as interindividual variability and condition-specific balance adaptations assessed through sensory organization test (SOT) and head-shake sensory organization test (HS-SOT) performance.

Previous studies examining exercise-based countermeasures during HDBR have reported mixed findings. While approaches such as resistance training combined with artificial gravity or fluid loading have demonstrated partial benefits in preserving specific physiological functions, they have not been sufficient to fully offset the adverse effects of prolonged inactivity^10–13^. A critical gap persists in maintaining balance control, a function particularly vulnerable to microgravity-induced deconditioning. These physiological alterations, including muscle atrophy, bone loss, and impaired postural stability, are well established in astronauts and closely parallel the changes observed during aging, underscoring the relevance of this model for both spaceflight and terrestrial health contexts. Aging naturally weakens muscles, bones, and balance systems, making older adults more susceptible to the same deconditioning effects during periods of extended inactivity, such as those observed in astronauts returning from space flights, including loss of muscle mass, bone density, and impaired balance^14–18^. Hence, studying exercise countermeasures in older adults can help identify strategies that may be applicable to astronauts, addressing shared challenges related to prolonged inactivity. This approach can also explore targeted physical training regimens for balance and mobility, especially in aging populations, to improve countermeasures for both groups^19,20^.

Among the various methods available to assess balance control, the SOT is commonly used to evaluate how visual, proprioceptive, and vestibular inputs contribute to postural stability. The addition of the head-shake condition has been shown to enhance the sensitivity of the SOT in identifying vestibular-related balance impairments^21,22^ as well as balance deficits in elderly individuals^23^. The HS-SOT challenges the postural control system by activating the vestibular system during head-shake movements, causing individuals to receive inaccurate sensory inputs. By superimposing head movements on body oscillations, the HS-SOT challenges the central nervous system to distinguish between vestibular inputs generated by head motion and those resulting from body sway. Individuals with vestibular dysfunction struggle to make this distinction, leading to increased body sway and reduced postural control^24–26^. As a result, the HS-SOT is valuable for identifying individuals with persistent vestibular deficits, even when their SOT performance appears near normal, as they might have developed compensations^27–30^. NASA uses the head-shake protocol in its training and recovery programs to simulate and assess astronauts’ vestibular systems and balance control in response to the sensory challenges encountered in space or post-spaceflight recovery^31–33^. The head-shake test thus helps detect vestibular or sensorimotor issues resulting from space exposure^21,22^. Consistent with this, findings from computerized dynamic posturography assessments in astronauts have shown that postflight balance impairments are most pronounced under conditions requiring active head movements and unstable support^33^. Dynamic head tilts increase fall incidence and prolong recovery, highlighting the sensitivity of vestibular-challenging tasks in detecting sensorimotor deficits following spaceflight.

Studies investigating the effects of HDBR on balance control have reported declines in postural stability that vary with the duration of bed rest^13,34–36^. Short-term interventions, such as a 14-day HDBR, have been shown to induce immediate postural instability, particularly in the medio-lateral axis, which largely recovered within four weeks^37^. Longer interventions, such as the 70-day HDBR, produced measurable deficits in standing balance that persisted up to 12 days post-intervention^9^. These findings suggest that both short- and long-duration HDBR can impact balance control, with longer durations generally producing more pronounced or prolonged effects. Moreover, evidence from spaceflight studies suggests that early exposure to progressively challenging sensorimotor tasks may facilitate recovery, although outcomes appear highly dependent on individual tolerance^38^. Movements that provoke vestibular symptoms may promote re-adaptation when performed within manageable thresholds, but excessive challenge can impair performance, highlighting substantial interindividual variability in recovery responses.

Building on these findings and considering the importance of vestibular-challenging tasks and individualized responses to sensorimotor stressors, the current study aims to evaluate the efficacy of specific physical training regimens in preserving balance control following a 2-week HDBR in healthy older adults. More specifically, the total daily exercise volume was distributed across three separate sessions. To our knowledge, this is the first study to investigate and apply this approach in this specific population. It is hypothesized that exercise countermeasures will significantly attenuate post-HDBR balance impairments, as measured by equilibrium score (ES) and strategy analysis (SA) on the SOT. Additionally, sex and age are anticipated to influence balance recovery, with potential differences in the magnitude of balance changes across these demographic factors. The study will also investigate the impact of the addition of the head-shake condition along the pitch axis (activating the anterior and posterior semi-circular canals), hypothesizing that it will better detect a change in balance control between exercise and control groups. Overall, this study investigates whether targeted physical training can mitigate HDBR-induced balance deficits and alter sensory integration during postural control.

## Results

### SOT and HS-SOT performance comparison between exercise and control groups

**Fig. 1:**
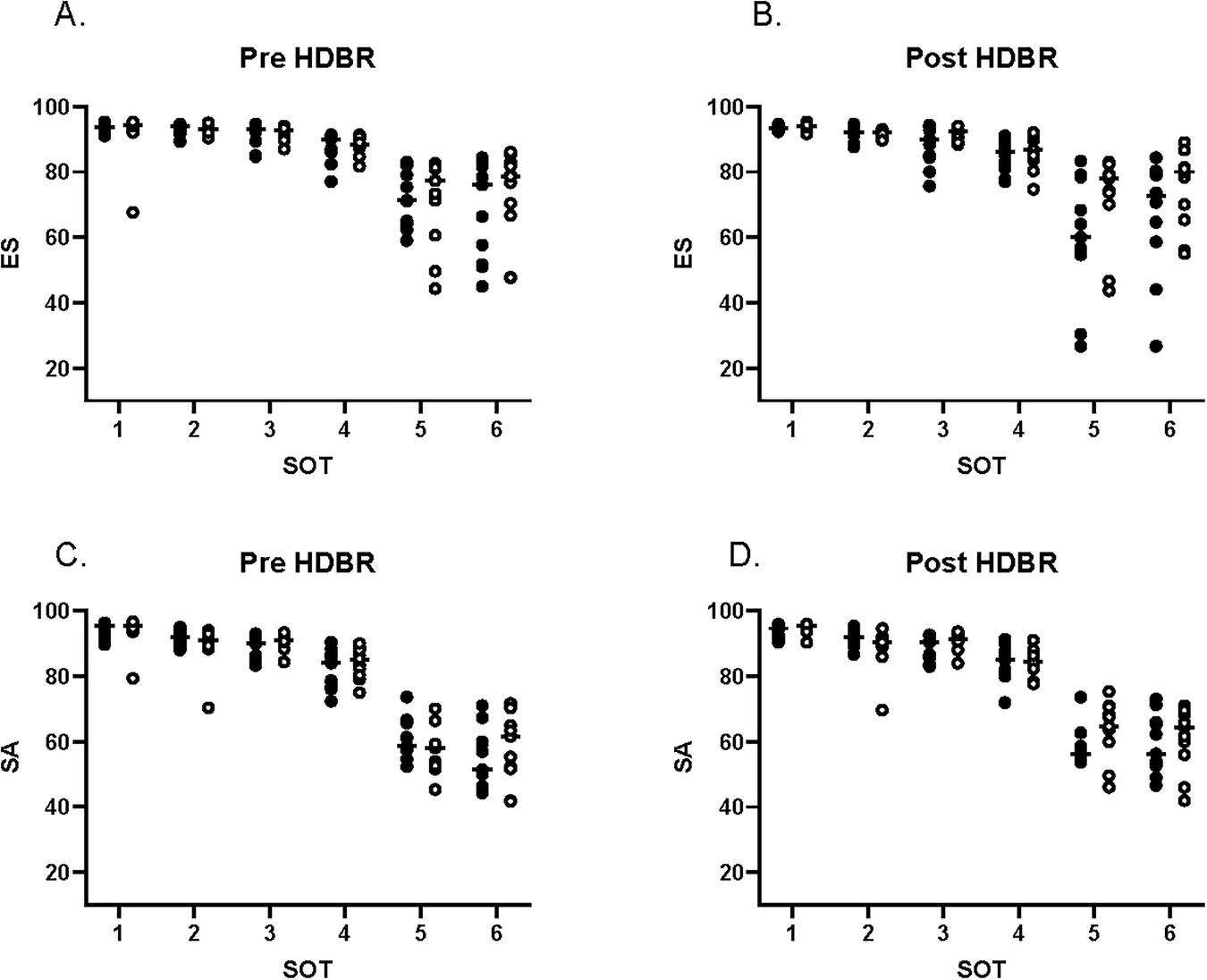
Equilibrium scores (ES) and strategy analysis scores (SA) for sensory organization test (SOT) conditions 1–6 in the control (CTRL, *n* = 11; ●) and exercise (EXE, *n* = 11; open circles) groups following 14-day head-down bed rest (HDBR). Individual participant values are shown for all panels. ES data are displayed in the top row: **A** pre-HDBR and **B** post-HDBR. SA data are displayed in the bottom row: **C** pre-HDBR and **D** post-HDBR.

**Fig. 2:**
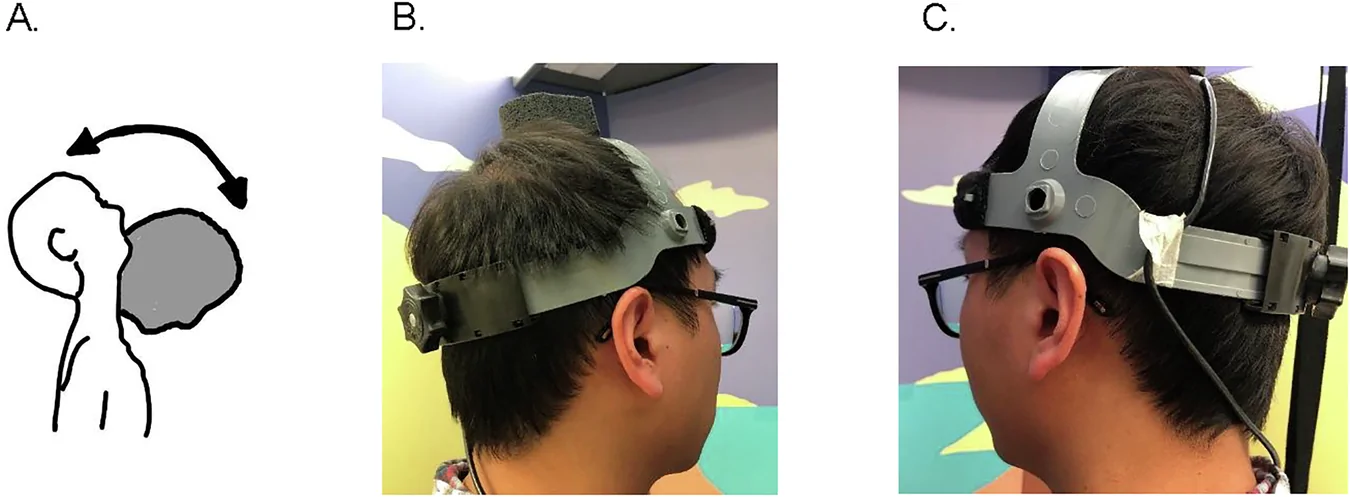
Illustration depicting head movement at 0.33 Hz and 20° amplitude for the head-shake sensory organization test (HS-SOT) conditions: SOT-2M (eyes closed, fixed surface) and SOT-5M (eyes closed, sway-referenced surface). **A** Schematic representation of head-shake stimulation (left panel). **B** Side view of a participant wearing the NeuroCom® head tracker. **C** Rear view of a participant wearing the NeuroCom® head tracker.

Balance performance was assessed using a computerized dynamic posturography system (NeuroCom), including the SOT and the HS-SOT. ES and SA were assessed pre- and post-HDBR in both exercise and control groups across six different SOT conditions. Head-shake conditions (SOT-2M and SOT-5M) were evaluated, with outcome measures including ES and vertical velocity, as well as derived ES ratios for fixed and sway conditions. Derived ES ratios were calculated to quantify changes in postural performance between fixed and sway conditions, expressed as the relative difference in ES between conditions with stable support versus conditions with induced sway, thereby providing an index of sensory reweighting under dynamic visual-vestibular demands. Table 1 presents the mean ± SD of the ES and SA for both groups under SOT and HS-SOT conditions. In both groups, pre-HDBR ES and SA decreased progressively from condition 1 to 6, reflecting increasing task difficulty (Fig. 1). Full descriptive statistics for all conditions (including SOT-2M and SOT-5M) are provided in Fig. 2.

**Table 1:** Mann–Whitney *U* test results for pre-HDBR equilibrium (ES) and strategy analysis (SA) across sensory organization test (SOT) conditions and with head-shake sensory organization test (HS-SOT), reported separately by group

Condition	Measure	Control group	Exercise group	Mann–Whitney *U*	*p*
1	ES	94.2 ± 2.2	93.8 ± 4.9	56.5	0.791
	SA	93.9 ± 1.3	91.6 ± 8.0	56.5	0.791
2	ES	91.6 ± 2.2	89.1 ± 6.5	46.0	0.340
	SA	93.4 ± 1.6	92.9 ± 1.4	43.0	0.248
3	ES	88.5 ± 3.8	90.5 ± 2.6	43.0	0.249
	SA	91.7 ± 3.7	91.8 ± 2.2	54.0	0.669
4	ES	82.0 ± 5.8	84.0 ± 4.6	49.0	0.450
	SA	87.4 ± 4.4	87.4 ± 3.1	52.5	0.598
5	ES	60.6 ± 6.1	57.9 ± 7.4	47.0	0.375
	SA	72.3 ± 8.8	71.2 ± 13.6	60.5	1.00
6	ES	54.3 ± 9.3	58.2 ± 10.4	46.0	0.341
	SA	69.1 ± 15.2	75.8 ± 11.0	46.0	0.341
ES ratio fixed	Score	0.97 ± 0.02	0.98 ± 0.24	48.5	0.425
ES ratio sway	Score	0.77 ± 0.18	0.94 ± 0.26	35.5	0.100
2M	Score	88.4 ± 2.3	89.5 ± 2.8	42.5	0.236
	Velocity	40.4 ± 9.8	39.4 ± 8.9	54.5	0.694
5M	Score	44.4 ± 12.8	52.1 ± 14.3	42.0	0.224
	Velocity	41.3 ± 11.0	38.5 ± 13.0	47.0	0.375

Velocity (deg/s). Values are means ± standard deviations.

*HS* head-shake, *Pre-HDBR* pre-head-down bed rest.

**Fig. 3:**
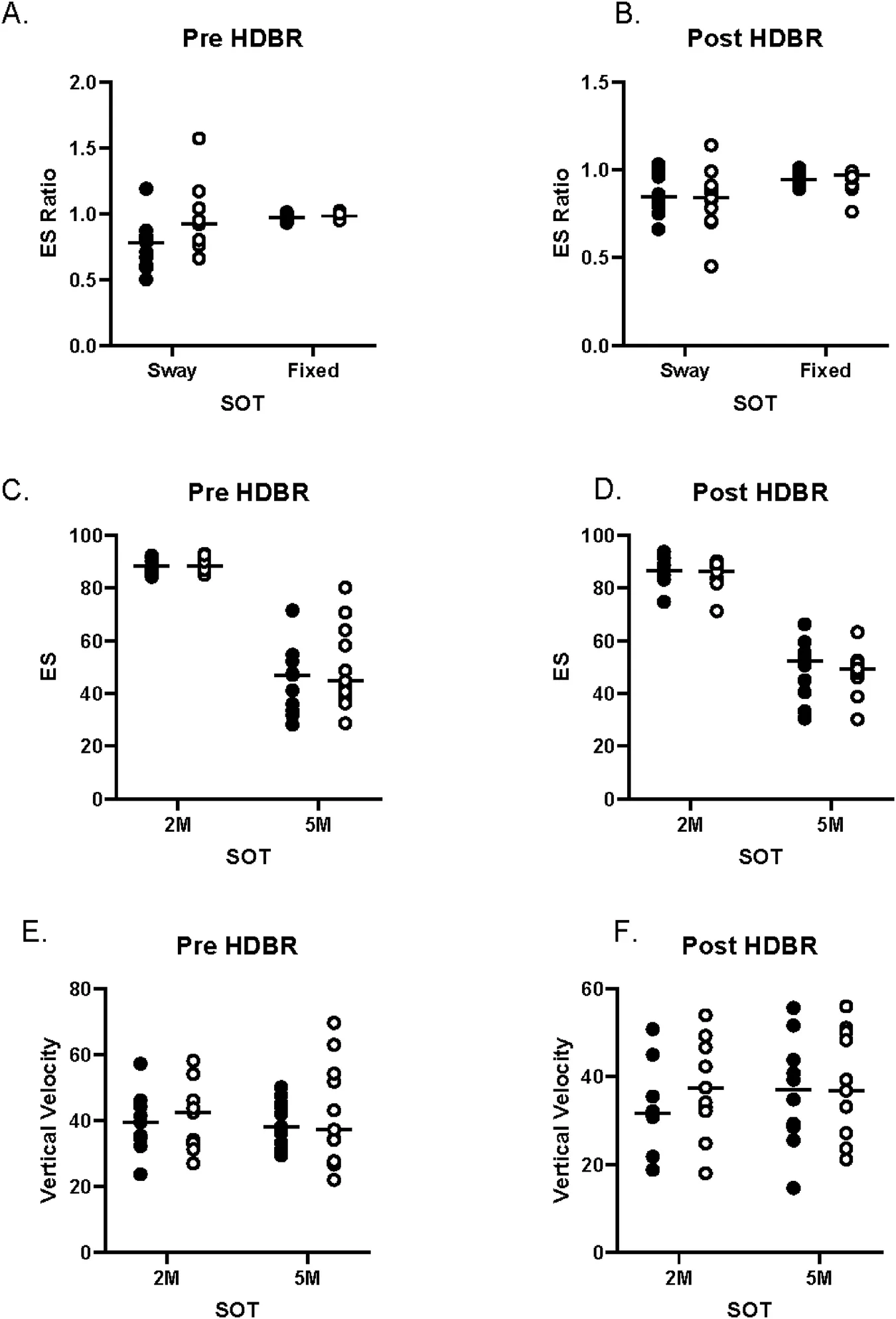
Equilibrium ratio (ES ratio) and outcomes from modified sensory organization test (SOT) conditions in the control (CTRL, *n* = 11; ●) and exercise (EXE, *n* = 11; ○) groups following 14-day head-down bed rest (HDBR). **A** ES ratio pre-HDBR (fixed vs. sway-referenced support; top row, left). **B** ES ratio post-HDBR (fixed vs. sway-referenced support; top row, right). **C** SOT-2M equilibrium score (SOT2M_ES) pre-HDBR (middle row, left). **D** SOT-2M equilibrium score post-HDBR (middle row, right). **E** SOT-2M vertical velocity (SOT2M_vv) pre-HDBR (bottom row, left); and **F** SOT-2M vertical velocity post-HDBR (bottom row, right). Data are presented as individual participant values.

In HS-SOT conditions, the pre-HDBR ES decreased from condition 2 to condition 5 for both control and exercise groups (Fig. 3C, D; middle panels), while head movement velocity (vertical velocity) remained similar between conditions (Fig. 3E, F). This indicates lower equilibrium scores under more challenging HS-SOT conditions.

At baseline, performance levels were similar between the two groups. Mann–Whitney *U* tests (independent samples) were conducted for each SOT and HS-SOT condition and confirmed no statistically significant differences in pre-HDBR ES or SA between the groups (*p* > 0.05 for all six conditions; Table 1 and see supplementary data for figures). After HDBR, no significant differences were found in ΔES or ΔSA across SOT conditions (*p* > 0.05; Table 2). For the HS-SOT, no significant group differences were observed in ES ratio fixed (*U* = 46.00, *p* = 0.337) or ES ratio sway (*U* = 32.00, *p* = 0.06). Similarly, no difference was observed in scores for HS-SOT conditions 2 (*U* = 44.50, *p* = 0.293) and 5 (*U* = 37.00, *p* = 0.123), nor in angular velocity for conditions 2 (*U* = 48.00, *p* = 0.411) and 5 (*U* = 50.00, *p* = 0.490; Table 2).

**Table 2:** Changes in score between pre- and post-HDBR conditions (ΔScore = Post−Pre)

Condition	Measure	Exercise group (ΔScore)	Control group (ΔScore)	Mann-Whitney *U* (EXE vs. CTRL)	*p*-value (EXE vs. CTRL)	Pooled data (pre vs. post; Sign Test, *p*, *n* = 22)
1	ES	1.82	−0.21	59.5	0.947	0.078
	SA	0.85	−18	59.0	0.921	1.00
2	ES	−1.21	−1.88	54.0	0.668	0.832
	SA	−0.39	0.18	49.0	0.447	0.027/N.S.
3	ES	−0.03	−3.39	58.0	0.869	0.664
	SA	0.42	0.18	37.0	0.119	0.286
4	ES	−1.3	−2.39	58.5	0.895	0.832
	SA	0.42	1.85	43.0	0.250	0.383
5	ES	0.85	−12.61	34.0	0.082	0.383
	SA	5.85	−1.97	33.5	0.076	0.832
6	ES	−0.36	−2.3	58.5	0.895	1.00
	SA	3.06	4.18	54.5	0.694	0.664
ES ratio	Fixed	−0.04	−0.02	46	0.337	0.383
	Sway	−0.10	0.08	32	0.06	0.286
2 M	ES	−4.0	−2.05	44.5	0.293	**<0.001**^‡^
	Velocity	−3.2	−4.78	48	0.411	0.286
5 M	ES	−2.32	3.07	37.0	0.123	0.134
	Velocity	−4.02	−2.10	50.0	0.490	0.134

Values are means ± standard deviations. ‡ Indicates a statistically significant between-group difference (*p* < 0.05) in change scores based on the Mann–Whitney *U* test or a significant post minus pre difference with the Sign Test. N.S. indicates that this comparison was no longer significant after statistical correction.

*HS* head-shake, *HDBR* head-down bed rest, *Pre* pre-HDBR, *Post* post-HDBR.

**Fig. 4:**
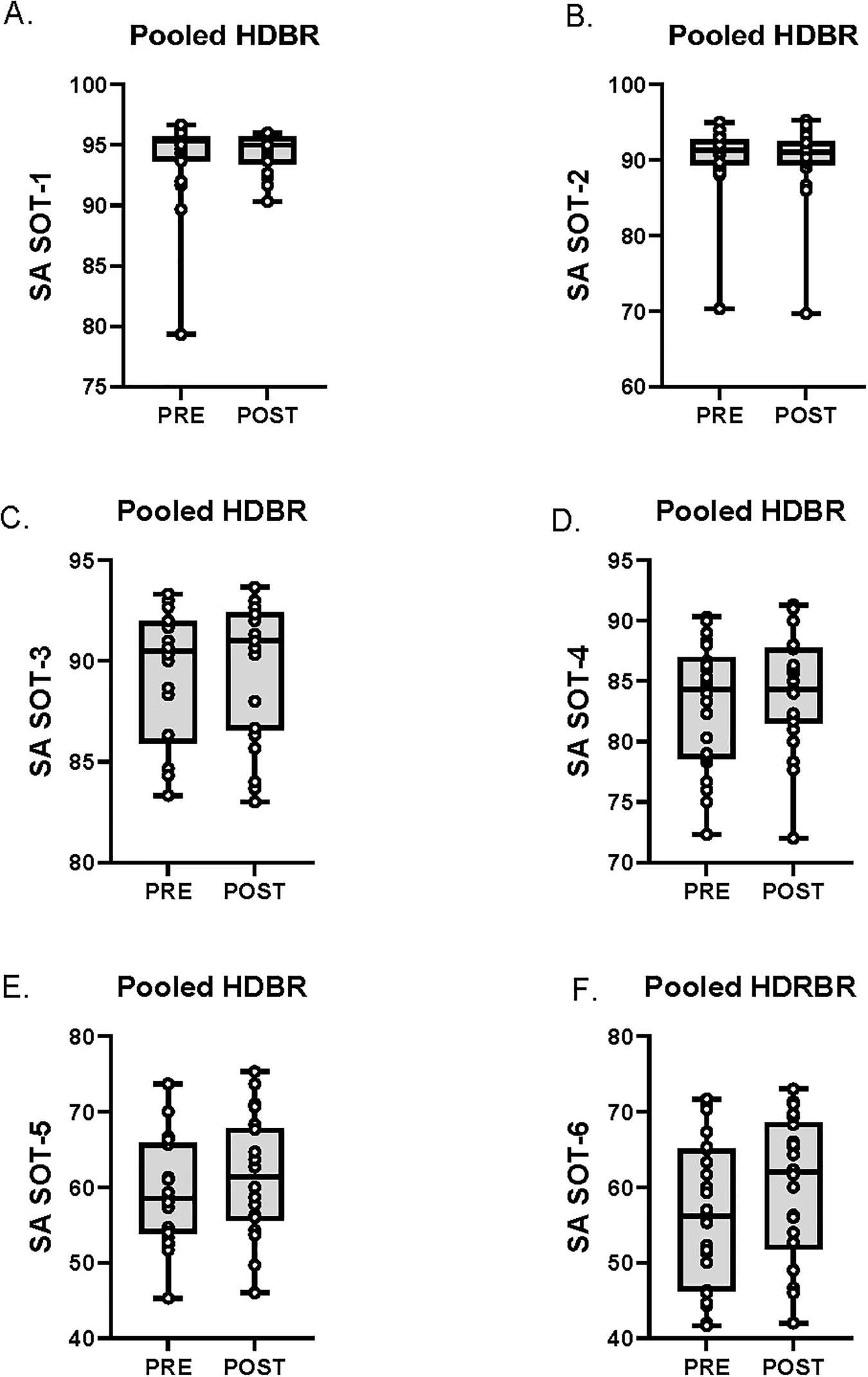
Box-and-whisker plots with overlaid individual data points showing strategy analysis score (SA) for pooled data across sensory organization test (SOT) conditions 1–6 in the exercise (EXE, *n* = 11) and control (CTRL, *n* = 11) groups before (Pre) and after (Post) 14-day head-down bed rest (HDBR). **A** SOT condition 1 Pre-Post HDBR (top left). **B** SOT condition 2 Pre-Post HDBR (top right). **C** SOT condition 3 Pre-Post HDBR (middle left). **D** SOT condition 4 Pre-Post HDBR (middle right). **E** SOT condition 5 Pre-Post HDBR (bottom left); and **F** SOT condition 6 Pre-Post HDBR (bottom right). Boxes represent the interquartile range (IQR) with the median indicated, whiskers denote the full range, and points correspond to individual participants. *Indicates a statistically significant between-group difference (*p* < 0.05) in change scores (Post−Pre), based on the Sign test.

**Fig. 5:**
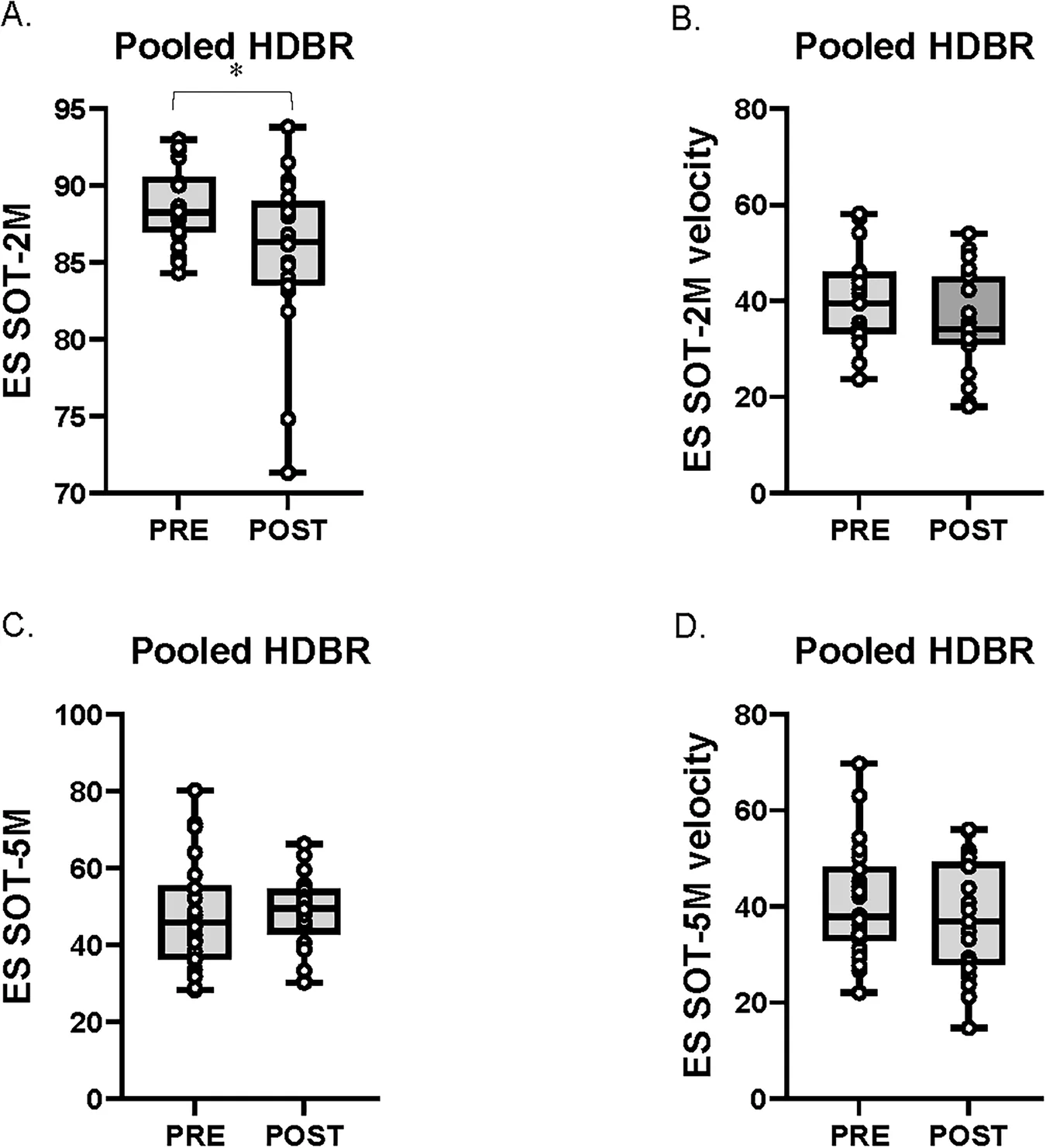
Box-and-whisker plots with overlaid individual data points showing equilibrium score (ES) and vertical velocity (vv) for head-shake conditions SOT-2M and SOT-5M before (Pre) and after (Post) 14-day head-down bed rest (HDBR). Panel **A** (top left) shows SOT-2M equilibrium score (ES) Pre- and Post-HDBR, panel **B** (top right) shows SOT-2M vertical velocity (vv) Pre- and Post-HDBR, panel **C** (bottom left) shows SOT-5M equilibrium score (ES) Pre- and Post-HDBR, and panel D (bottom right) shows SOT-5M vertical velocity (vv) Pre- and Post-HDBR. Boxes represent the interquartile range (IQR) with the median indicated, whiskers denote the full range, and points correspond to individual participants. *Indicates a statistically significant difference in change scores (Post−Pre) based on the Sign test (*p* < 0.001).

When all subjects were pooled together, a significant pre–post HDBR difference was observed for ∆SA in SOT condition 2 (*p* = 0.027; Table 2; pooled data column; Fig. 4). However, it was no longer observed after Holm–Sidak corrections and no significant pre–post differences were found for ∆SA in the other five conditions or in the ∆ES (all *p* > 0.05). For the HS-SOT, a significant pre–post HDBR difference was detected for SOT-2M using the Sign Test (*p* < 0.001; Table 2; Fig. 5), but not for SOT-5M. No significant pre–post HDBR differences were observed for angular velocity in either condition.

### Effects of pre-HDBR

Spearman correlations were conducted for each SOT and HS-SOT condition to examine the relationship between pre-HDBR ES and SA and their respective changes (ΔES and ΔSA), to assess whether baseline performance was associated with subsequent changes following HDBR (see Table 3).

**Table 3:** Spearman correlation coefficients (*ρ*) between pre-HDBR scores and change scores (ΔScore = Post–Pre) for change scores across sensory organization test (SOT) conditions, are reported separately by group and pooled for equilibrium scores (ES) and strategy analysis (SA)

Condition	Measure	Control group (*n* = 11; *ρ*)	Exercise group (*n* = 11; *ρ*)	Pooled data from all participants (*n* = 22; *ρ*)
1	ES	−0.55	**−0.71**†	**−0.67**†
	SA	**−0.85**†	**−0.66**†	**−0.81**†
2	ES	−0.02	−0.29	−0.13
	SA	0.15	**−0.84**†	−0.39
3	ES	0.01	−0.06	0.01
	SA	−0.19	**−0.77**†	−0.39
4	ES	−0.47	**−0.73**†	**−0.61**†
	SA	−0.32	−0.53	**−0.44**†
5	ES	−0.26	−0.20	−0.24
	SA	0.13	−0.38	−0.10
6	ES	−0.25	−0.55	−0.35
	SA	−0.53	−0.28	**−0.49**†
ES ratio	Fixed	**−0.651†**	−0.114	−0.377
	Sway	−0.117	**−0.733†**	**−0.547†**
2M	ES	−0.06	−0.05	−0.09
	Velocity	−0.46	−0.33	−0.40
5M	ES	−0.46	**−0.77**†	**−0.66**†
	Velocity	−0.10	−0.51	**−0.48†**

Values are means ± standard deviations. Bold values followed by † indicate a statistically significant Spearman correlation between pre-HDBR scores and change scores (ΔScore = Post–Pre) (*p* < 0.05) either within groups (Control or Exercise) or when data of all participants are pooled together.

*HS* head-shake, *HDBR* head-down bed rest, *Pre* pre-HDBR, *Post* post-HDBR.

**Fig. 6:**
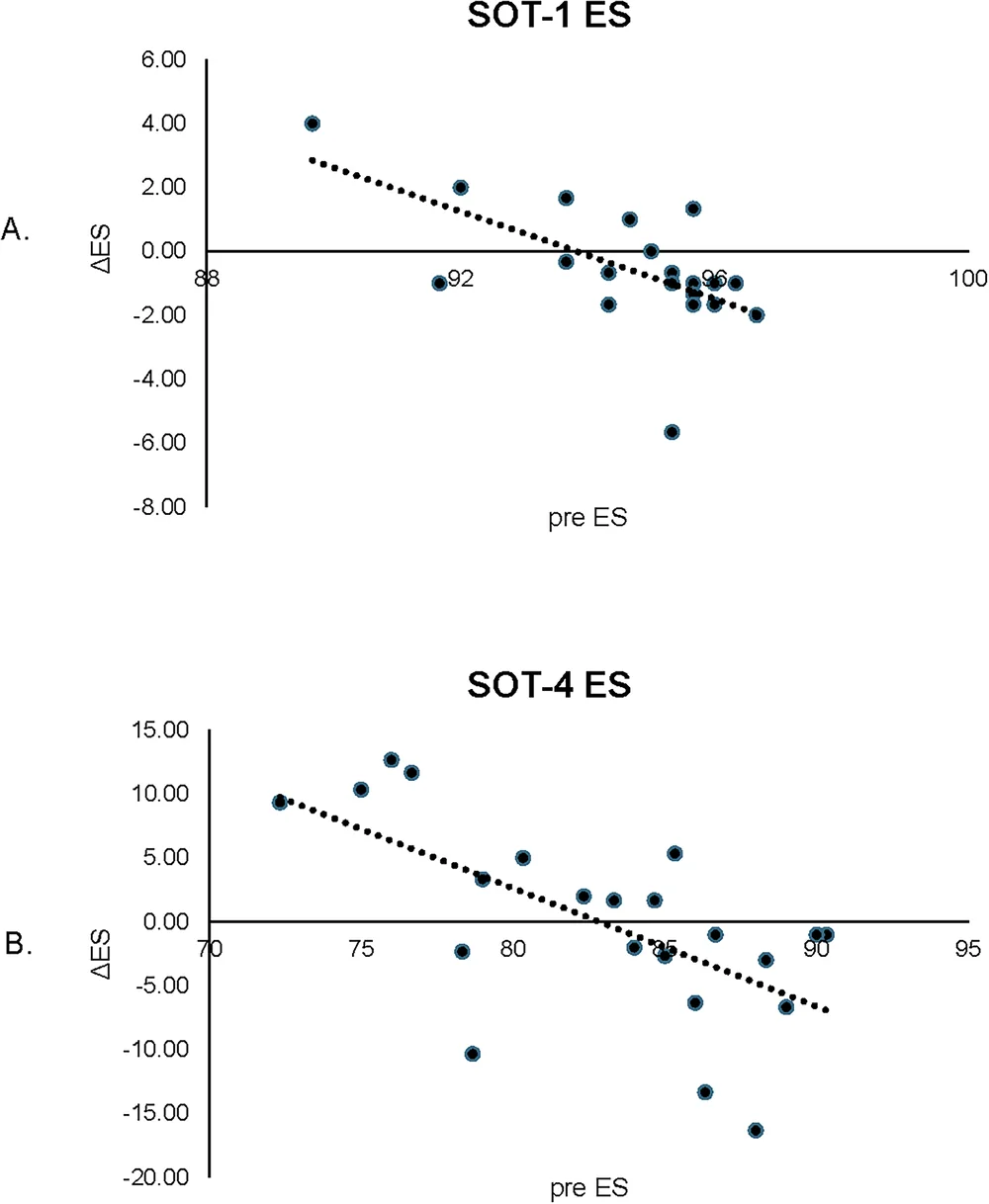
Correlation between pre–head-down bed rest (HDBR) equilibrium score (ES) and change in ES (ΔES) in pooled participants (*n* = 22). Points represent individual participants. The *x*-axis shows pre-ES, and the *y*-axis shows ΔES. Dotted lines indicate group-specific linear regression fits. For sensory organization test (SOT) conditions 1 and 4, significant correlations between pre ES and ΔES were observed (Spearman’s rank-order correlation, *p* < 0.05). **A** SOT condition 1 (top panel) and **B** SOT condition 4 (bottom panel).

For the ES, pre-HDBR ES showed a significant negative correlation with ∆ES in the exercise group in the SOT-condition 1 (*R* = -0.71, *p* = 0.014) and SOT-condition-4 (*R* = −0.73, *p* = 0.011). No correlation was observed in the control group. When both groups were pooled together, a significant negative correlation was also observed in SOT-condition 1 (*R* = −0.67; *p* < 0.001) and SOT-condition 4 (*R* = −0.61; *p* = 0.002), suggesting that baseline ES prior to HDBR was associated with the magnitude of change following HDBR, irrespective of group allocation (Fig. 6).

**Fig. 7:**
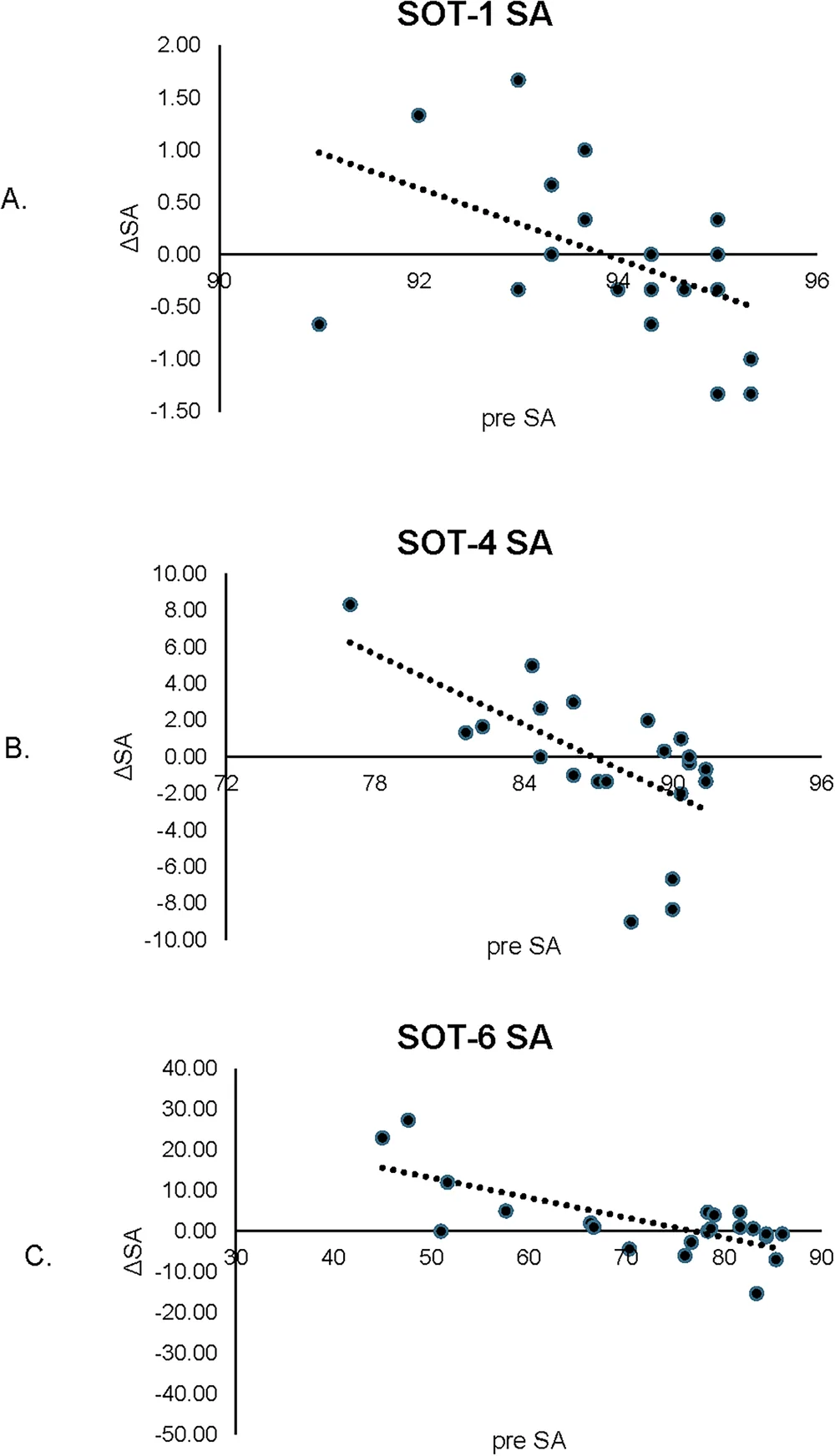
Correlation between pre–head-down bed rest (HDBR) strategy analysis score (SA) and change in SA (ΔSA) in pooled participants (*n* = 22). Points represent individual participants. The *x*-axis shows pre-SA, and the *y*-axis shows ΔSA. Dotted lines indicate group-specific linear regression fits. Significant correlations between pre-SA and ΔSA were observed for sensory organization test (SOT) conditions 1, 4, and 6 (Spearman’s rank-order correlation, *p* < 0.05). **A** SOT condition 1 (top panel), **B** SOT condition 4 (middle panel), and **C** SOT condition 6 (bottom panel).

For the SA, pre-HDBR SA exhibited significant negative correlation with ∆SA in SOT-condition 1 in the control group (*R* = −0.85, *p* = 0.001) and in the exercise group (*R* = −0.66, *p* = 0.026). The exercise group showed significant negative correlation in condition 2 (*R* = −0.84, *p* = 0.001) and condition 3 (*R* = −0.77, *p* = 0.006). When data for both groups were pooled, significant correlations were found in condition 1 (*R* = −0.81, *p* < 0.001), condition 4 (*R* = −0.44, *p* = 0.042), and condition 6 (*R* = −0.49, *p* = 0.02; Fig. 7).

**Fig. 8:**
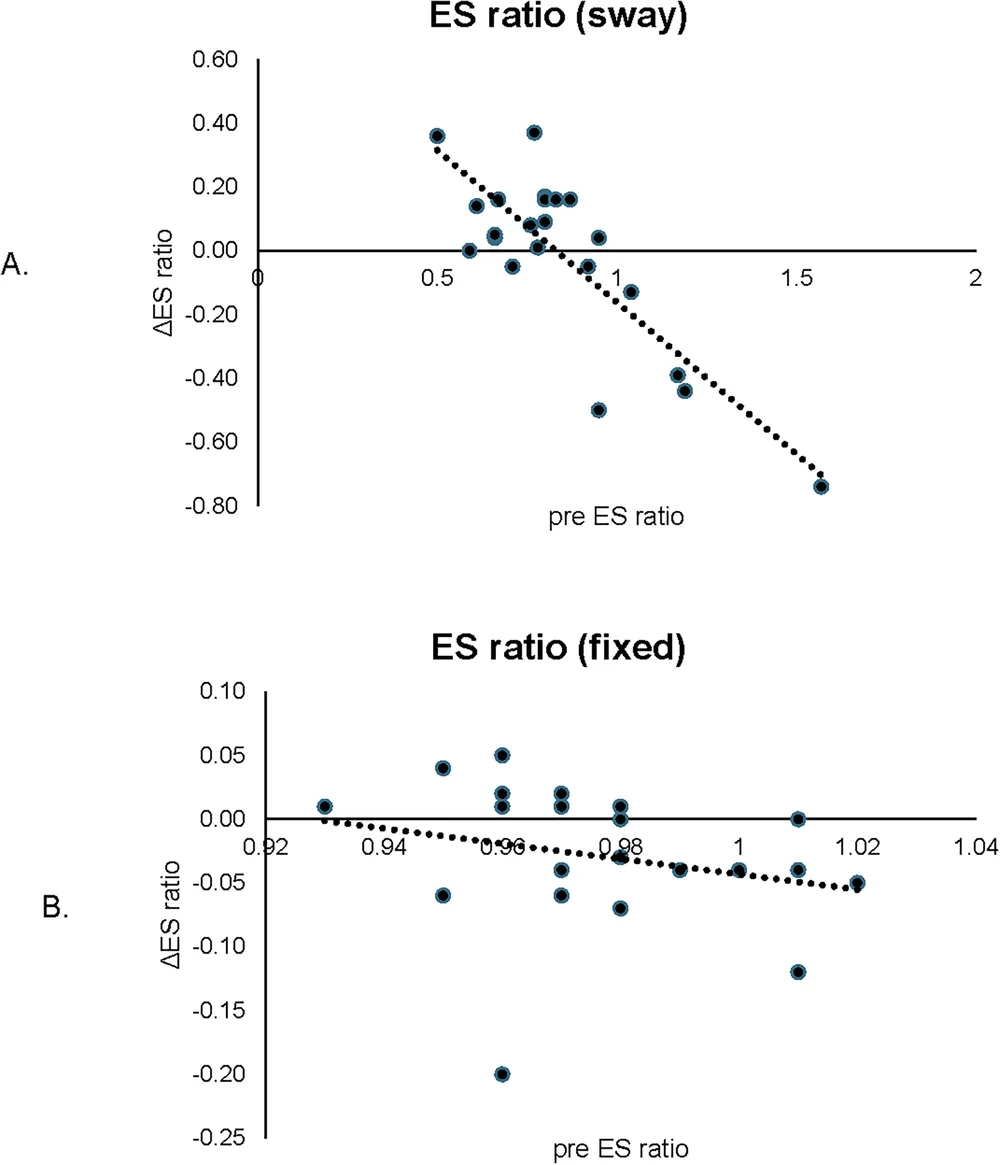
Relationship between pre-head-down bed rest (HDBR) equilibrium score (ES) ratio and change in ES ratio (ΔES ratio) in pooled participants (*n* = 22) under sway-referenced and fixed support conditions. Points represent individual participants. The *x*-axis shows the pre-HDBR ES ratio, and the *y*-axis shows the ΔES ratio. Dotted lines indicate group-specific linear regression fits. Significant correlations between pre-HDBR ES ratio and ΔES ratio were observed in both conditions (Spearman’s rank-order correlation, *p* < 0.05). **A** Sway-referenced condition (top panel) and **B** fixed support condition (bottom panel).

**Fig. 9:**
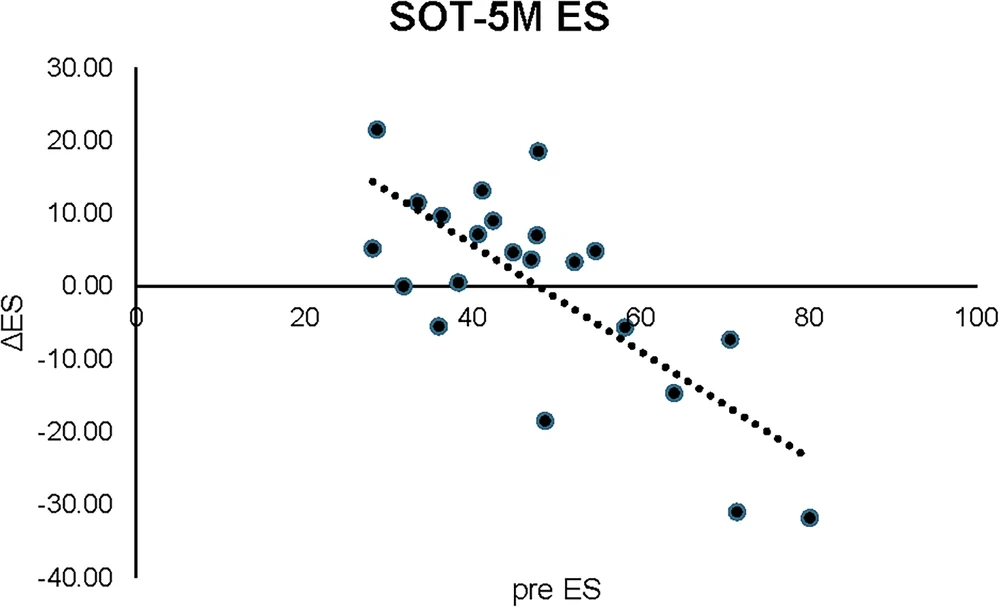
Correlation between pre-head-down bed rest (HDBR) equilibrium score (ES) and change in ES (ΔES) in pooled participants (*n* = 22). Points represent individual participants. The *x*-axis shows pre-HDBR ES, and the *y*-axis shows ΔES. The dotted line indicates the linear regression fit for the pooled data. A significant correlation between pre-HDBR ES and ΔES was observed for the sensory organization test (SOT) SOT-5M (head-shake condition) (Spearman’s rank-order correlation, *p* < 0.05).

In HS-SOT conditions, a significant correlation was found between ES ratio fixed and baseline performance in the control group (*r* = −0.651; *p* = 0.03), and between ES ratio sway and baseline performance in the exercise group only (*r* = −0.733; *p* = 0.01). Furthermore, in the exercise group, there was a significant correlation between pre-HDBR SOT-5M and ΔES during SOT-5M (*R* = −0.77, *p* = 0.005), whereas no significant correlation was observed for angular velocity (*R* = −0.51; *p* = 0.109). In the control group, no significant correlation was found for ∆ES (*R* = −0.46 *p* = 0.160) or angular velocity (*R* = −0.10, *p* = 0.77). When data were pooled, a significant negative correlation was observed between pre-HDBR ES ratio sway and ∆ES ratio sway (*R* = −0.547; *p* = 0.008; Fig. 8), as well as between pre-HDBR SOT-5M and ΔES during SOT-5M (*R* = −0.47; *p* = 0.026; Fig. 9).

All these correlations suggest that individuals with higher pre-HDBR performance exhibited smaller changes (including negative changes) following HDBR, whereas individuals with lower pre-HDBR performance showed greater changes in balance performance.

### Effects of age and sex on SOT performance

To evaluate whether participant age affected the ∆ES or ∆SA, Spearman correlations were conducted using the pooled data across both groups (Table 4). No significant correlations were found between age and ∆ES or ∆SA in the pooled data or when analyzed separately within the exercise and control groups (*p* > 0.05). The influence of participant sex on ∆ES or ∆SA was assessed using Mann–Whitney *U* tests for each SOT condition. No statistically significant sex differences were observed in the pooled sample or within individual groups (*p* > 0.05).

**Table 4:** Spearman correlation coefficients between (*ρ*) Age and ∆Scores, and Mann–Whitney *U* Tests (*U*) for sex differences in ∆Scores for stability (ES) and strategy analysis (SA) across SOT conditions (Pooled Data)

Condition	Measure	Age (*n* = 22)		Sex (*n* = 22)	
		Spearman *ρ*	*p*	Mann-Whitney *U*	*p*
1	ES	−0.07	0.774	46.5	0.354
	SA	−0.12	0.584	59.0	0.921
2	ES	−0.08	0.736	42.5	0.235
	SA	−0.10	0.659	51.0	0.530
3	ES	−0.24	0.278	55.5	0.742
	SA	-0.16	0.492	49.5	0.466
4	ES	−0.21	0.355	44.0	0.278
	SA	−0.26	0.245	59.5	0.948
5	ES	−0.04	0.850	50.0	0.491
	SA	−0.27	0.231	44.0	0.278
6	ES	0.09	0.688	60.0	0.974
	SA	0.04	0.850	56.5	0.793
ES ratio	Fixed	−0.008	0.972	60	0.974
	Sway	0.149	0.508	41.5	0.211
2 M	ES	−0.03	0.914	50.0	0.490
	Velocity	0.14	0.546	45.0	0.308
5 M	ES	0.06	0.795	54.0	0.670
	Velocity	−0.28	0.208	49.5	0.470

*HS* head-shake, *n/a* no data.

In HS-SOT conditions, age and sex were examined in relation to ES ratio (fixed and sway), ∆ES during SOT-2M and SOT-5M, and angular velocity. Spearman correlations showed no significant relationships between age in either variable (*p* > 0.05). Mann–Whitney *U* tests revealed no significant sex differences in either of these scores (*p* > 0.05).

## Discussion

The aim of this study was to evaluate the effectiveness of applied exercises as countermeasures to prevent or limit detrimental changes in balance control following a 14-day HDBR period. An important novel aspect of this study is that the prescribed daily exercise volume was distributed across three sessions rather than performed as a single continuous bout, and that it was studied in people 50 years and older. The initial hypothesis was that the participants in the exercise group who regularly participated in physical training would maintain their postural stability after HDBR. To test this hypothesis, the ES and SA derived from SOT, as well as the HS-SOT, were used to evaluate the ability to integrate visual, somatosensory, and vestibular information to maintain balance under various conditions. The results show that: (1) Based on the score from the SOT (ES and SA) and the HS-SOT measures (ES ratio, SOT-2M and SOT-5M), there was no difference in the ability to maintain balance between the exercise and control groups after HDBR; (2) When participants from both groups were pooled, a significant effect of bed rest was observed for the SA in the SOT-condition 2 and for the SOT-2M. This suggests that the postural strategy used by participants to maintain quiet stance with their eyes closed without (SOT-condition 2) and with head-shake (SOT-2M) was altered after HDBR; (3) Initial balance performance pre-HBDR (ES, SA, ES ratio and SOT-5M) was associated with the magnitude of change in balance control following HDBR; (4) No effect of age and sex was detected on SOT or HS-SOT scores. Taken together, these results suggest that the applied countermeasures during the 2-week HDBR did not modify postural control under challenging sensory conditions. These results suggest that intrinsic individual factors, such as baseline balance abilities, influence responses to 2-week HDBR.

Two-week HDBR did not influence the ability to maintain postural stability in either exercise or control groups. However, when both groups were pooled, a significant effect of HDBR on postural strategies (SA) was observed during quiet stance with eyes closed (SOT-condition 2) and during head-shake conditions (SOT-2M). These observations are consistent with studies reporting declines in postural control after spaceflight durations as short as 4 days^2,39^, although those studies primarily report differences in SOT-conditions 5 and 6^40^.

One explanation for this discrepancy might lie in the HDBR model itself. Although 6° HDBR is a well-established analog for simulating the effects of microgravity, it has limitations: while it effectively reproduces cephalad fluid shifts, it does not eliminate gravitational load^41^. Notably, previous studies using HDBR for 42–63 days did not show changes in SOT-condition 1 through 5^4^, and a duration of 70 days of HDBR was required to observe changes in the SOT-5M^36^, which includes head movements during eyes-closed perturbation^42^.

Importantly, the present findings suggest that conditions involving either reduced visual reliance (SOT-condition 2) or combined sensory and head-movement demands (SOT-2M) may be particularly sensitive to subtle changes in postural strategy following HDBR. Both conditions involve distinct sensory processing demands: SOT-condition 2 relies primarily on vestibular and somatosensory integration in the absence of visual input, whereas SOT-2M additionally introduces vestibular perturbation through head movements. This may indicate that HDBR-related adaptations are more readily expressed in conditions where sensory reweighting is required rather than in purely visually dominant or more complex multi-conflict conditions.

Furthermore, these results are aligned with evidence suggesting that impairments in postural strategy are a key feature of post-spaceflight balance alterations^39^. While longer HDBR duration may provide additional insight into the effects of physical countermeasures, studies using extended bed rest periods have similarly reported limited changes in ES, in contrast to post-spaceflight observations.

It has been suggested that the magnitude of the effects of microgravity exposure and the recovery time depend not only on the duration of exposure but also on interindividual differences in the physiological adaptation^6,43^. Because participants’ baseline physical conditions vary (also see Koppelmans et al., 2022), this may influence their ability to maintain balance. To explore this relationship, Spearman correlation was conducted as an exploratory analysis to examine the association between pre-HDBR ES and SA and their respective changes (∆ES or ∆SA) following HDBR.

The results revealed significant negative correlations (Table 3). In the exercise group, these correlations were significant for ES in SOT-condition 1 and 4, for SA in SOT-condition 1–3, and for SOT-5M. In the control group, a significant correlation was observed only in SOT-condition 1. When data were pooled, significant correlations were found in SOT-conditions 1 and 4 for both ES and SA, as well as in SOT-condition 6 and in SOT-5M for SA.

The interpretation of these correlations is not straightforward, as these associations are not independent. The association between pre-HDBR ES and SA and their respective changes (∆ES or ∆SA) was primarily observed during (a) quiet standing with eyes open (SOT-condition 1), (b) backward perturbation with eyes open (SOT-condition 4), and (c) eyes-closed conditions with head-shake during perturbations (SOT-5M). These conditions reflect differing levels of sensory integration demands, ranging from minimal sensory conflict (SOT-condition 1) to altered somatosensory input (SOT-condition 4), and combined sensory system changes (SOT-5M). The absence of similar associations in more complex HS-SOT and SOT-5/6 conditions may reflect greater interindividual variability, task-specific noise, or potential ceiling/floor effects that reduce the sensitivity in these measures.

Overall, a negative association between balance performance and change following the 2-week HDBR period was observed across participants, irrespective of group allocation. However, this relationship should be interpreted with caution; it may be influenced by mathematical coupling and the regression to the mean inherent in change score analysis. Although exploratory subgroup analyses suggested a strong pattern in the exercise group, these findings are descriptive and not indicative of a group-specific effect.

Given these limitations, the observed association should not be interpreted as evidence that baseline performance determines responsiveness. While interindividual differences may reflect underlying physiological variability (e.g., neuromuscular control, muscle function, and skeletal properties)^44,45^, the present analysis does not allow these mechanisms to be directly inferred.

No significant difference in ES or SA was observed between men and women, nor was there an effect of age on balance. However, given the relatively small sample size, the study may not have been sufficiently powered to detect such effects. Evidence from the literature indicates that physiological aging is associated with declines in sensory sensitivity and perception, degeneration of the neuromuscular system, and reductions in muscle and bone strength^46^. Notably, participants in this study were aged 55–65 years, whereas these factors are more strongly associated with postural instability and increased fall risk in the population over 65 years of age^46,47^.

One limitation of this study lies in the inter-individual variability of factors that could influence the observed results, such as age and participants’ initial balance abilities. Accordingly, the impact of the HDBR period may vary depending on individual intrinsic characteristics, rather than being fully captured by group-based analyses. Moreover, recovery following spaceflight can occur rapidly, often within approximately 4 days^39^. As noted in the Methodology, some participants were unable to perform the balance assessment immediately after the HDBR. This delay may have led to an overestimation of post-HDBR ES and SA relative to the immediate effect of bed rest.

In addition, the stability limits set at 12.5° by the NeuroCom® Balance Master are theoretical values and may not reflect true individual limits. Some participants may exceed these values, meaning that ES and SA may not fully represent their actual balance capacity. Another limitation relates to repeated testing using the NeuroCom® system. Repeated exposure to the SOT and HS-SOT may induce learning or practice effect^48,49^, which are more pronounced in the elderly population^50^. Although performance generally declines following HDBR, such learning effects may have partially offset these declines and could explain improvements observed in some individuals. Furthermore, while the test–retest reliability of the HS-SOT has been reported as moderate to good, performance is influenced by the plane of head movement, with pitch-plane movements proving particularly challenging^50^. These factors should be considered when interpreting the results.

In conclusion, the findings of this study suggest that the applied countermeasures had limited effectiveness in mitigating the adverse effects of microgravity simulated by 2-week HDBR on balance control. Additionally, intrinsic individual factors, such as baseline balance performance, may be associated with responses to HDBR.

## Methodology

### Study design overview

All participants provided written informed consent prior to participation. The study was approved by the Research Ethics Board of the McGill University Health Centre (MUHC; study no. 2021-7170) and conducted in accordance with the Declaration of Helsinki. This study was a two-arm randomized controlled trial in which participants were allocated to either an exercise or a control group. Participants and investigators were not blinded to group assignment. Participants resided at the McConnell Centre for Innovative Medicine (CIM), Research Institute of the McGill University Health Centre for 26 days, including a 5-day baseline, 14-day head-down bed rest (HDBR), and 7-day recovery phase. Recruitment began in July 2021, with data collection spanning baseline, HDBR, and recovery phases, and the study was conducted from July 2021 to early 2022. A detailed description of study procedures, including measurements, intervention, and recovery protocols, is provided elsewhere^51^. This trial was registered at ClinicalTrials.gov (NCT04964999).

### Randomization

Participants were randomly assigned to either 14 days of 6° head-down bed rest (HDBR) alone (control) or HDBR with an exercise countermeasure (exercise group). The random allocation sequence was generated using a computer-based procedure by a study team member not involved in recruitment, enrollment, or outcome assessment. Restricted randomization was used to maintain balance between groups, with stratification by sex where applicable. The block size was not disclosed to the recruitment or assessment team to preserve allocation concealment. The allocation sequence was kept separate from personnel involved in enrollment and baseline testing, and group assignment was revealed only after completion of screening and baseline procedures.

Due to the nature of the intervention, participants and exercise supervisors could not be blinded to group allocation. However, outcome assessors and data analysts were blinded to group assignment whenever feasible. To minimize bias, standardized testing procedures were applied across groups. Both groups followed the same 14-day 6° HDBR protocol, hospital environment, monitoring procedures, and testing schedule, with the exercise intervention as the only difference.

### Participant demographics

A total of 23 participants were enrolled in this study. One participant dropped out on day 3 of HDBR, mainly for convenience reasons (hard to stay in position, difficulties to pass bowel movement, and required assistance for basic needs). Two participants were withdrawn from the study on day 3 of recovery due to the occurrence of atrial fibrillation. However, in this manuscript, data from these 2 participants are included in the analysis since the balance and NeuroCom® measurements were conducted on day 1 of recovery. Sample size was based on feasibility considerations and the logistical constraints of conducting a controlled head-down bed rest study^51,52^. All participants followed a standardized diet described in the methodology paper^51^ based on their energy requirements measured using an indirect calorimeter. Participants’ baseline characteristics are shown in Table 5 and standard measures of the Canadian Space Agency are published in a separate manuscript^52^.

**Table 5:** Participants characteristics at baseline

	Control	Exercise
*n*	11	11
Sex	F (5), M (6)	F (6), M (5)
Age (y)	58.4 ± 3.9	58.4 ± 3.4
Height (cm)	166.7 ± 10.7	167.1 ± 8.4
Weight (kg)	67.5 ± 14.9	72.4 ± 13.3
BMI (kg/m^2^)	24.0 ± 2.8	25.7 ± 2.9
Systolic blood pressure (mmHg)	123 ± 16	128 ± 9
Diastolic blood pressure (mmHg)	76 ± 12	77 ± 5
Total lean mass (kg)	45.9 ± 10.7	47.8 ± 11.7
Total fat mass (kg)	19.3 ± 5.5	21.9 ± 54.0
Lower limb Muscle strength (N)	124.0 ± 48.3	130.1 ± 41.7
V̇O_2peak_ (ml/kg/min)	30.2 ± 6.1	32.0 ± 6.9
Glucose (mmol/l)	4.7 ± 0.4	4.8 ± 0.2

### Eligibility criteria

Participants were healthy adults aged 55–65 years. Inclusion criteria were non-smokers; body mass index between 20 and 30 kg/m²; height between 158 and 190 cm; engagement in ≥2.5 h per week of moderate-to-vigorous aerobic physical activity; and successful completion of medical and psychological screening. Female participants were required to be postmenopausal (≥1 year since last menses or documented ovariectomy, with serum FSH > 30 IU/L). Participants were required to be willing to be randomly assigned to either the exercise or control group.

Exclusion criteria included cardiovascular, neurological, vestibular, or musculoskeletal disorders that could affect balance or participation in head-down bed rest (HDBR), including a history of myocardial infarction, arrhythmias, thrombosis risk, orthostatic intolerance, seizures, or significant head trauma. Additional exclusion criteria were electrocardiogram abnormalities, anemia, low bone mineral density, renal or gastrointestinal disorders, chronic back pain, migraines, severe allergies, or mental illness. Participants were also excluded if they required medications that could interfere with study outcomes, had a recent poor nutritional status, claustrophobia, special dietary restrictions, or metallic implants. Further exclusions included smoking within 6 months, substance abuse within 30 days, blood donation within 3 months, or participation in another study within 2 months prior to enrollment.

### Exercise intervention details

Briefly, participants in the exercise group performed three daily sessions, alternating between HIIT and low-intensity cycling, and resistance exercises targeting upper and lower body musculature. The total daily exercise duration ranges from 60–75 min. All exercise routines were meticulously executed either in a head-down tilt or horizontal orientation, utilizing designated equipment based on a structured timetable. Crucially, the intensity levels of these exercise interventions were tailored to individual participants, aligning with their performance capabilities and tolerances. HIIT sessions consisted of repeated bouts of high-intensity effort interspersed with recovery periods, while continuous aerobic sessions were performed at moderate intensity. Resistance training included multi-joint exercises targeting major muscle groups. Participants in the control group did not perform structured exercise but received standard physiotherapy care, consisting of passive range-of-motion exercises to maintain joint mobility. A comprehensive description of the intervention protocol, including exercise parameters and progression, is available in ref. ^53^.

### Instrumentation

**Fig. 10:**
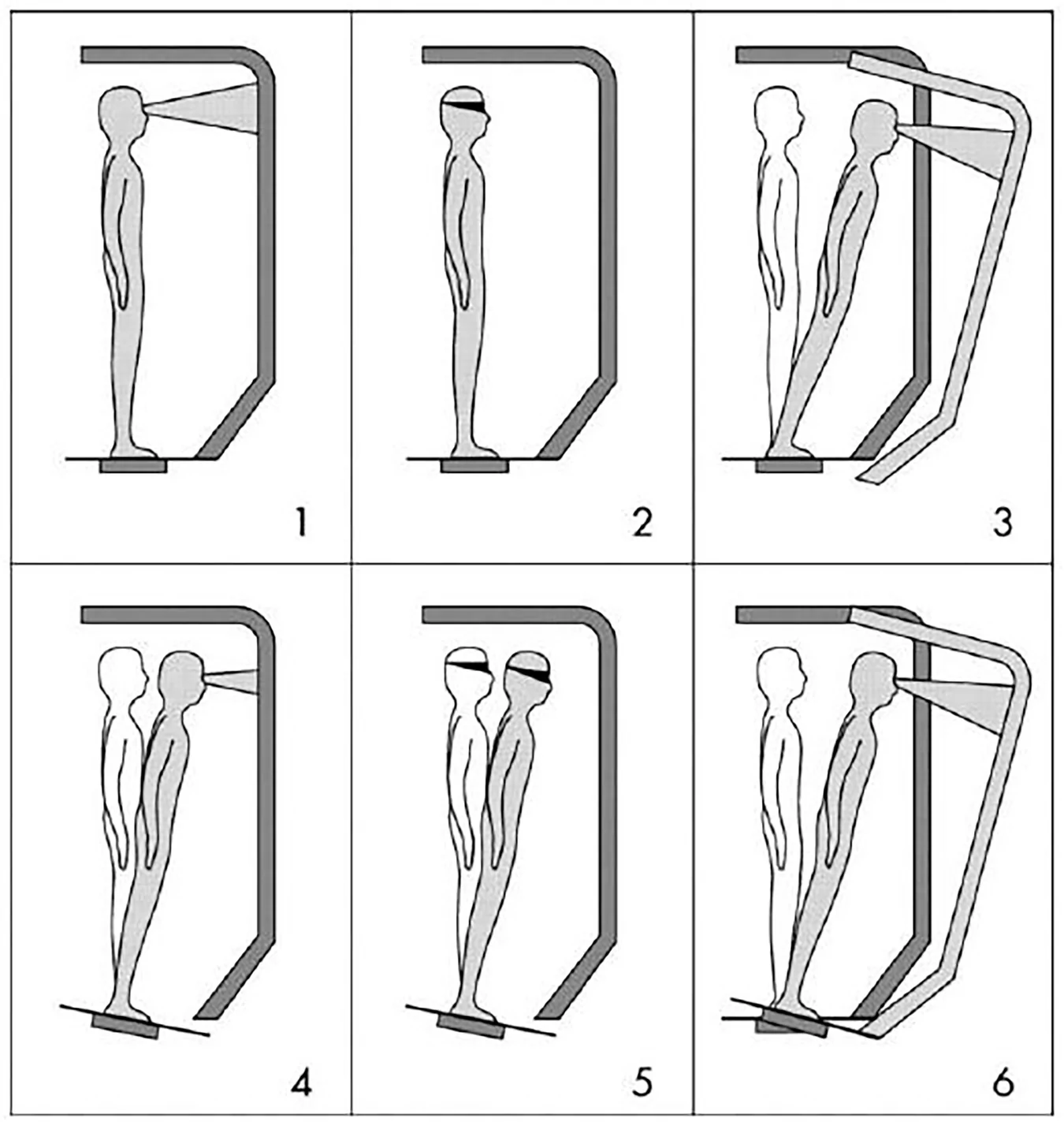
Sensory organization test (SOT) conditions (adapted from NeuroCom® SMART EquiTest® computerized dynamic posturography). The six conditions are presented in a 2 × 3 layout (top row: conditions 1–3; bottom row: conditions 4–6): (1) eyes open, stable support and stable surrounding; (2) eyes closed, stable support; (3) eyes open, stable support with sway-referenced surrounding; (4) eyes open, sway-referenced support with stable surrounding; (5) eyes closed, sway-referenced support; (6) eyes open, sway-referenced support and surrounding. In the present study, participants stood with arms crossed over the chest during testing.

Data collection was performed using the NeuroCom® Balance Master™ SMART EquiTest® (Clackamas, OR), which is equipped with the Computerized Dynamic Posturography system. With its standardized tests, this system, which is included in the NeuroCom® Balance Master™ SMART EquiTest® device, has been identified as an effective method for assessing the ability to maintain a stable position and identify abnormalities in the use of different sensory systems^54^. It has been used widely in research to quantify postural stability^55,56^. This device includes a safety harness, a force platform, and a moving visual environment that oscillates in the sagittal plane. Among the dynamic posturography protocols, the Sensory Organization Test (SOT; Fig. 10) was applied to the participants before and after the HDBR period.

Standing postural balance was selected as the primary balance outcome because the objective of the study was to evaluate sensory integration and postural control under standardized conditions following HDBR. The SOT and HS-SOT allow controlled manipulation of visual, somatosensory, and vestibular inputs, making it possible to identify condition-specific changes in balance performance and postural strategy. These tests are also widely used in spaceflight and HDBR research to assess sensorimotor deconditioning and recovery.

### Sensory organization test (SOT)

In this study, the SOT was used to assess participants’ ability to integrate sensory components for balance maintenance under different conditions. The test consists of six conditions, applied in a specific order (as illustrated in Table 6), with participants required to stand on the platform in a standard position with arms crossed over their chest. Figure 10 also illustrates the different conditions, as suggested by NeuroCom®. However, in the current study, the participants were standing with arms crossed, which differs from that shown in Fig. 10. Participants were instructed to maintain balance for 20 seconds during three consecutive trials of each condition^4^.

**Table 6:** Variation of parameters for each SOT condition

Condition	Vision	Visual field	Force plate	Perturbed/eliminated system	Reliable system
1	Eyes open	Stable	Horizontal	None	Vestibular; Vision; Somatosensory
2	Eyes closed	Absent	Horizontal	Vision eliminated	Vestibular; Somatosensory
3	Eyes open	Unstable	Horizontal	Vision perturbed	Vestibular; Somatosensory
4	Eyes open	Stable	Unstable	Somatosensory perturbed	Vestibular; Vision
5	Eyes closed	Absent	Unstable	Somatosensory perturbed; Vision eliminated	Vestibular
6	Eyes open	Unstable	Unstable	Somatosensory and vision were perturbed	Vestibular

In Condition 1, balance was assessed with the participant’s eyes open and no perturbation of the platform. In Condition 2, participants stood on the platform with their eyes closed. In Condition 3, participants had their eyes open while the cabin was tilted, but the platform remained stable. In these conditions (2 and 3), visual cues were either absent or inaccurate, requiring the participant to prioritize proprioceptive and vestibular information for balance maintenance. In Condition 4, participants had their eyes open, and the surrounding environment remained stable, while the platform was sway-referenced and tilted to perturb proprioceptive information from the ankles. In Condition 5, the platform was tilted with the eyes closed, resulting in the absence of visual input and perturbation of proprioceptive cues. In Condition 6, the eyes were open, but both the visual surround and support surface moved simultaneously. This configuration created a situation where both visual and proprioceptive cues were inaccurate, forcing the brain to rely solely on vestibular signals to restore balance^55^. Each of these conditions was performed three times, the resulting ES and SA were averaged together for the mean and SD value.

The primary aim of the SOT was to selectively isolate one sensory cue by creating sensory conflicts, allowing the evaluation of the brain’s ability to rely on the most reliable sensory input to maintain body orientation and balance^54^. These conditions provided insight into which sensory system might be more affected following the HDBR period, which could have implications for postural control in microgravity or other altered environments^54^.

In addition to the six standard conditions, two SOT-2M and SOT-5M conditions were specifically tested, where participants performed continuous chin-to-chest nod along the pitch axis movements at a frequency of 0.33 Hz and an amplitude of 20° in each direction. In SOT-2M (Eyes Closed, Fixed Surface), participants stood on a fixed surface without visual input, relying on proprioceptive and vestibular feedback. In SOT-5M (Eyes Closed, Sway-Referenced Surface), participants stood on a sway-referenced surface with the platform moving in response to postural sway, while visual input was absent. Each of these conditions was performed six times, with data collected continuously at 0.33 Hz head rotation. One un-scored practice trial was completed for each condition, followed by three scored trials, each lasting 15 s. The tests followed a preset order and repetition to ensure consistency in data collection^31,32^. White noise was supplied to mask auditory cues, and participants’ arms were folded across their chests. Participants performed both the standard and head-moving trials (SOT-2M and SOT-5M) before and after the HDBR period. Post-HDBR, participants were assessed on the 1st day of verticalization (*n* = 15), or, if participants experienced dizziness after verticalization, on the 2nd day (*n* = 5) or 3rd day (*n* = 2) after verticalization.

### Outcome measures: SOT-equilibrium score (ES)

Following the six conditions of the SOT, different scores were generated by NeuroCom®. For each trial, ES was calculated as a percentage based on the participants’ anteroposterior sway amplitude relative to theoretical stability limits^57^. These stability limits were defined as 12.5° of anteroposterior sway to represent the boundaries of upright stability^57^. ES ranges from 0 to 100, with higher values indicating better postural stability^58^. Scores decrease as the participants approached anterior or posterior limits (8.5° anterior and 4° posterior thresholds)^57^. A score of 0 was assigned when the participants stepped off the support surface or exceeded the maximum stability limit^58^. ES reflects the integrated contribution of visual, proprioceptive, and vestibular systems in maintaining upright posture^59^.

### Outcome measures: SOT-strategy analysis (SA)

The ankle and hip strategies consist of postural reactions involving activation of muscles acting at the ankle and hip joints, respectively, to stabilize the upright standing balance following a perturbation. The ankle strategy is the first line of postural reaction when the perturbation is perceived as small. When the perturbation is perceived as larger or more threatening to balance, the hip strategy can be used.

The quantification of ankle or hip strategies^60,61^ is achieved using SA, which is derived by combining the data from the force plate and ES. SA, ranging from 0 to 100, was computed by the NeuroCom® system for each condition. SA reflects the degree of ankle and hip strategies used throughout each trial to maintain postural stability. When participants adjusted their balance primarily through ankle movements, SA approached 100. Conversely, if hip movements were the predominant strategy, SA approached 0. The resulting values between 0 and 100 are the result of combining the two strategies^62^.

### Outcome measures: HS-SOT—equilibrium score and angular velocity

In the HS-SOT conditions, only ES were computed by the NeuroCom® system. SA were not available for these trials. As in the standard SOT, ES reflected participants’ postural stability based on their sway relative to the antero-posterior stability limits.

In addition to ES, the angular velocity of the head movement was monitored during the head-shake conditions to ensure consistency across trials and participants. Head movements were performed along the pitch axis (anteroposterior), and angular velocity values were compared between conditions to confirm that task execution was similar. This step helped ensure that any differences in postural performance were attributable to sensory integration demands rather than variability in head movement dynamics.

Thirdly, the ES ratio was calculated to quantify the relative change in postural stability between head-shake and head-fixed conditions. The overall ES ratio was defined as the mean ES of the head-shake conditions (SOT-2M and SOT-5M) divided by the mean ES of the corresponding head-fixed conditions (SOT-2 and SOT-5). To distinguish between support conditions, condition-specific ratios were also computed. The ES ratio fixed was defined as the ratio of SOT-2M to SOT-2, representing the fixed support condition. The ES ratio sway was defined as the ratio of SOT-5M to SOT-5, representing the sway-referenced support condition.

### Statistical analysis

Statistical analysis was conducted using IBM SPSS Statistics 28.0 or 29.0 (IBM Corp., Armonk, NY) or SAS 9.4 (SAS Institute Inc., Cary, NC) software. Descriptive statistics, including participants’ sex, mean age, and height, are presented as mean and standard deviation (for continuous variables) or frequencies for categorical variables. This study followed a 2-arm randomized controlled design, with participants allocated to either an exercise or control group. Analyses were conducted according to this grouping structure, with repeated measurements obtained pre- and post-HDBR across SOT and HS-SOT conditions. Some analyses used pooled data where the group was not considered. Missing data were not imputed, and analyses were conducted using available data at each time point.

Given the non-normal distribution of most variables and the relatively small sample size, nonparametric tests were used for all inferential analyses. At baseline, Mann-Whitney tests were used to confirm group comparability on outcome measures (ΔES and ΔSA). For the principal analysis, Mann-Whitney tests were performed on the post–pre differences (ΔES and ΔSA; defined as post-HDBR–pre-HDBR) to assess between-group differences in response to HDBR across all SOT and HS-SOT conditions. When no between-group differences were observed in ΔES or ΔSA, Sign tests were applied to the pooled sample to assess overall pre-post effects. When differential group evolution was observed, Sign tests were performed separately for each group. The Sign test was preferred over the Wilcoxon test because several variables did not meet the assumption of symmetric distributions required for the Wilcoxon test. To account for multiple comparisons, Holm–Šidák corrections were applied within families of related tests. Specifically, corrections were performed separately for between-group comparisons (Mann–Whitney *U* tests) and pooled within-sample analyses (Sign tests). Statistical significance was set at *p* < 0.05.

Spearman’s rank-order correlations were conducted to assess the relationships between baseline (pre-HDBR) performance (ES, SA, ES ratio, and HS-SOT outcomes) and change scores (ΔES, ΔSA, and ΔES ratio), as well as between age and change scores. Mann–Whitney *U* tests were also used to assess sex differences in ΔES and ΔSA. These analyses were performed across all participants and within each group (exercise and control).

## Supplementary information

Supplementary materials_April 29, 2026-Final

## Data Availability

Data is provided within the manuscript or supplementary information files.
