# Supplementary material for: The impact of 14-day head-down bed rest with or without an exercise countermeasure on standing balance control: a randomized controlled trial
Source: NPJ Microgravity. 2026 Jun 26;12:87. doi: 10.1038/s41526-026-00624-x (PMC13672788; doi:10.1038/s41526-026-00624-x)
Supplement: Supplementary file 1 [file 41526_2026_624_MOESM1_ESM.pdf]

Supplementary materials

Title: The impact of 14-Day Head-Down Bed Rest with or without an Exercise countermeasure on Standing Balance Control

Nok-Yeung Law[1,2], Karima Ahmed Yahia[1], Joseph Gill-Lussier[1,2], Ali Falaki[1], Helga Tonkov[1], Faezeh Abbariki[1,2], Andréa Faust[3], José A. Morais[3,4], Guy Hajj-Boutros*[3], Dorothy Barthélemy*[1,2]

[1] NeuroMobility Lab, Institut Universitaire de Réadaptation en Déficience Physique de Montréal, site of Center for Interdisciplinary Research in Rehabilitation of Greater Montreal (CRIR), CIUSSS du Centre-Sud-de-l'Ile-de-Montréal, QC, Canada.
[2] School of Rehabilitation, Faculty of Medicine, Université de Montréal, Montreal, QC, Canada.
[3] Research Institute of McGill University Health Centre, McGill University, Montréal, QC, Canada
[4] Division of Geriatric Medicine, McGill University Health Centre, McGill University, Montréal, QC, Canada

* Shared Corresponding author

Dorothy Barthélemy, PhD, MSc, PT
School of Rehabilitation, Faculty of Medicine
Université de Montréal
Pavillon du Parc,
C.P.6128 Succ. Centre-ville
Montreal Qc, Canada, H3C 3J7
Phone: (514) 343-7712 Fax: (514) 343-6929
E-mail: dorothy.barthelemy@umontreal.ca

Guy Hajj-Boutros, PhD(c)
Research Institute of McGill University Health Centre
McGill University
Montréal, QC, Canada, H4A 3J1
Phone : (514) 9340-1934
Email : guy.elhajjboutros@mail.mcgill.ca

Running title: Bedrest and balance control

Keywords: Bedrest, Older adult, Aerobic exercise, Exercise countermeasure

Table S1. Mean ± Standard Deviation (SD)

Measures	Control (n = 11)		Experimental (n = 11)		Total (CTL + Exp, n = 22)	
	pre	post	pre	post	pre	post
ES condition 1	94.2 ± 2.2	93.8 ± 1.3	93.8 ± 4.9	94.1 ± 2.0	94.0 ± 3.7	94.0 ± 1.6
ES condition 2	91.6 ± 2.2	91.2 ± 2.5	89.1 ± 6.5	88.5 ± 6.3	90.4 ± 4.9	89.8 ± 4.9
ES condition 3	88.5 ± 3.8	88.4 ± 5.9	90.5 ± 2.6	91.0 ± 3.1	89.5 ± 3.3	89.7 ± 4.8
ES condition 4	82.0 ± 5.8	82.8 ± 6.3	84.0 ± 4.6	82.8 ± 5.8	82.8 ± 5.9	83.0 ± 5.2
ES condition 5	60.6 ± 6.1	59.3 ± 9.2	57.8 ± 7.4	62.4 ± 11.3	59.2 ± 6.8	60.9 ± 10.2
ES condition 6	54.3 ± 9.3	57.2 ± 16.4	58.2 ± 10.4	61.8 ± 12.6	56.3 ± 9.8	59.5 ± 14.5
SA condition 1	93.9 ± 1.2	93.7 ± 0.8	91.6 ± 8.0	93.5 ± 1.2	93.6 ± 1.0	92.8 ± 5.7
SA condition 2	93.5 ± 1.6	91.5 ± 2.5	92.9 ± 1.4	91.7 ± 1.1	91.6 ± 1.9	93.1 ± 1.5
SA condition 3	91.7 ± 3.7	88.3 ± 6.3	91.8 ± 2.2	91.7 ± 1.9	90.0 ± 4.9	91.7 ± 3.0
SA condition 4	87.4 ± 4.4	85.0 ± 7.8	87.4 ± 3.1	86.1 ± 5.3	85.6 ± 4.9	87.4 ± 3.8
SA condition 5	72.3 ± 8.8	59.7 ± 18.4	71.2 ±13.6	72.1 ± 13.9	65.9 ± 17.1	71.8 ± 11.2
SA condition 6	69.1 ± 15.2	66.8 ± 17.6	75.8 ± 11.0	75.4 ± 12.0	71.1 ± 15.4	72.4 ± 13.4
ES ratio fixed	0.97 ± 0.02	0.95 ± 0.04	0.98 ± 0.02	0.94 ± 0.07	0.84 ± 0.15	0.85 ± 0.23
ES ratio sway	0.77 ± 0.18	0.84 ± 0.13	0.94 ± 0.26	0.83 ± 0.17	0.97 ± 0.22	0.94 ± 0.05
SOT-M2 (score)	88.4 ± 2.3	86.2 ± 5.0	89.5 ± 2.8	85.3 ± 5.4	85.8 ± 5.1	89.0 ± 2.6
SOT-M2 (velocity)	40.4 ± 9.8	35.0 ± 10.0	39.4 ± 8.9	37.7 ± 10.7	36.3 ± 10.2	39.9 ± 9.2
SOT-M5 (score)	44.4 ± 12.8	49.1 ± 10.9	52.1 ± 14.3	48.3 ± 8.5	48.7 ± 9.6	48.3 ± 13.8
SOT-M5 (velocity)	41.3 ± 11.0	37.8 ± 12.6	38.5 ± 13.0	37.5 ± 11.2	37.6 ± 11.7	39.9 ± 11.9

Table S2. SOT Condition 1 (Equilibrium Score)

Group	Sex	Age	Participant number	Pre-bed rest			Post-bed rest		
				Test 1	Test 2	Test 3	Test 1	Test 2	Test 3
Control	F	56	PT04	92	96	93	88	95	97
	M	61	PT26	94	97	96	93	94	95
	F	55	PT22	96	96	97	95	94	97
	M	66	PT27	93	93	89	89	92	91
	M	64	PT38	90	94	85	96	90	95
	M	59	PT43	95	96	95	94	95	95
	F	57	PT56	94	97	96	94	96	94
	F	57	PT68	94	90	92	96	92	94
	M	56	PT69	95	96	97	95	95	95
	M	59	PT73	96	96	95	93	94	96
	F	58	PT79	94	95	94	94	91	93
Experimental	M	57	PT21	92	94	97	93	94	94
	M	57	PT36	94	95	92	95	94	97
	M	57	PT39	96	96	95	96	97	98
	F	59	PT49	95	94	97	95	87	87
	F	58	PT50	97	95	95	91	95	96
	F	64	PT52	94	96	96	95	93	95
	M	61	PT54	52	92	94	85	95	95
	F	63	PT62	96	96	96	94	95	94
	F	60	PT64	93	96	95	95	97	95
	F	60	PT65	96	95	94	96	95	94
	M	58	PT75	96	97	97	94	96	94

Table S3. SOT Condition 2 (Equilibrium Score)

Group	Sex	Age	Participant number	Pre-bed rest			Post-bed rest		
				Test 1	Test 2	Test 3	Test 1	Test 2	Test 3
Control	F	56	PT04	94	94	94	94	95	94
	M	61	PT26	89	87	91	90	86	90
	F	55	PT22	94	95	96	95	96	93
	M	66	PT27	91	90	93	85	89	91
	M	64	PT38	91	80	93	87	91	89
	M	59	PT43	93	90	89	90	93	86
	F	57	PT56	93	92	93	92	94	94
	F	57	PT68	84	91	93	92	90	83
	M	56	PT69	94	91	91	90	95	93
	M	59	PT73	90	93	94	92	94	95
	F	58	PT79	92	93	95	92	91	89
Experimental	M	57	PT21	91	86	88	84	87	88
	M	57	PT36	91	89	94	93	94	94
	M	57	PT39	90	92	91	85	89	91
	F	59	PT49	89	91	89	87	88	88
	F	58	PT50	93	92	90	93	90	88
	F	64	PT52	87	91	90	88	88	93
	M	61	PT54	88	93	88	93	94	88
	F	63	PT62	92	95	95	94	90	85
	F	60	PT64	92	95	89	87	95	95
	F	60	PT65	94	92	93	92	92	94
	M	58	PT75	71	72	68	69	71	72

Table S4. SOT Condition 3 (Equilibrium Score)

Group	Sex	Age	Participant number	Pre-bed rest			Post-bed rest		
				Test 1	Test 2	Test 3	Test 1	Test 2	Test 3
Control	F	56	PT04	92	92	91	94	93	93
	M	61	PT26	88	75	90	84	87	92
	F	55	PT22	94	92	92	94	95	94
	M	66	PT27	74	92	88	71	85	81
	M	64	PT38	88	92	90	90	84	89
	M	59	PT43	82	95	77	87	82	95
	F	57	PT56	92	93	91	94	96	96
	F	57	PT68	74	88	88	84	81	81
	M	56	PT69	89	89	93	91	95	95
	M	59	PT73	94	94	91	86	94	94
	F	58	PT79	73	93	93	71	82	87
Experimental	M	57	PT21	87	86	92	86	91	90
	M	57	PT36	92	92	92	92	95	95
	M	57	PT39	94	91	93	90	94	91
	F	59	PT49	85	94	92	88	88	86
	F	58	PT50	83	95	95	89	91	93
	F	64	PT52	78	81	94	77	89	93
	M	61	PT54	86	94	92	86	92	91
	F	63	PT62	95	94	87	92	88	86
	F	60	PT64	89	93	94	91	96	95
	F	60	PT65	81	92	93	92	93	94
	M	58	PT75	94	93	93	95	96	97

Table S5. SOT Condition 4 (Equilibrium Score)

Group	Sex	Age	Participant number	Pre-bed rest			Post-bed rest		
				Test 1	Test 2	Test 3	Test 1	Test 2	Test 3
Control	F	56	PT04	81	88	89	78	85	76
	M	61	PT26	66	76	86	84	91	91
	F	55	PT22	71	80	84	79	74	75
	M	66	PT27	68	83	85	48	80	77
	M	64	PT38	74	85	93	79	81	86
	M	59	PT43	70	93	91	86	81	92
	F	57	PT56	86	86	88	84	82	91
	F	57	PT68	86	93	92	89	89	90
	M	56	PT69	48	81	88	71	82	92
	M	59	PT73	70	78	82	85	87	93
	F	58	PT79	87	91	87	85	87	84
Experimental	M	57	PT21	72	91	87	86	86	83
	M	57	PT36	71	90	86	77	86	90
	M	57	PT39	84	90	81	81	83	83
	F	59	PT49	73	89	75	83	85	79
	F	58	PT50	75	77	89	87	84	85
	F	64	PT52	89	91	87	74	85	88
	M	61	PT54	84	85	90	60	81	78
	F	63	PT62	79	95	90	74	76	65
	F	60	PT64	77	77	71	85	83	88
	F	60	PT65	88	92	90	82	91	94
	M	58	PT75	74	91	91	91	91	90

Table S6. SOT Condition 5 (Equilibrium Score)

Group	Sex	Age	Participant number	Pre-bed rest			Post-bed rest		
				Test 1	Test 2	Test 3	Test 1	Test 2	Test 3
Control	F	56	PT04	67	53	56	52	56	49
	M	61	PT26	52	50	70	43	64	68
	F	55	PT22	58	66	76	46	68	58
	M	66	PT27	50	58	67	48	38	46
	M	64	PT38	45	72	67	30	67	67
	M	59	PT43	70	60	67	49	70	82
	F	57	PT56	61	61	50	57	66	70
	F	57	PT68	72	81	68	72	77	72
	M	56	PT69	58	62	63	63	70	74
	M	59	PT73	43	56	65	40	53	50
	F	58	PT79	51	67	39	62	73	57
Experimental	M	57	PT21	59	68	72	65	66	64
	M	57	PT36	40	52	68	71	67	64
	M	57	PT39	43	47	65	26	30	47
	F	59	PT49	40	55	67	81	51	66
	F	58	PT50	25	69	80	77	67	68
	F	64	PT52	66	66	46	68	51	65
	M	61	PT54	46	32	58	59	59	57
	F	63	PT62	27	63	68	60	38	58
	F	60	PT64	52	67	59	68	67	61
	F	60	PT65	62	70	78	65	77	75
	M	58	PT75	63	64	72	76	67	79

Table S7. SOT Condition 6 (Equilibrium Score)

Group	Sex	Age	Participant number	Pre-bed rest			Post-bed rest		
				Test 1	Test 2	Test 3	Test 1	Test 2	Test 3
Control	F	56	PT04	30	36	67	44	19	53
	M	61	PT26	1	66	72	81	75	82
	F	55	PT22	47	48	55	66	19	37
	M	66	PT27	56	56	68	34	45	60
	M	64	PT38	11	63	64	71	75	65
	M	59	PT43	56	80	66	50	81	64
	F	57	PT56	54	42	75	70	75	85
	F	57	PT68	60	73	80	61	71	65
	M	56	PT69	37	48	49	43	75	68
	M	59	PT73	30	68	56	16	26	48
	F	58	PT79	49	66	63	33	62	68
Experimental	M	57	PT21	53	84	78	51	61	73
	M	57	PT36	55	55	47	24	66	76
	M	57	PT39	57	55	54	71	71	69
	F	59	PT49	42	24	60	54	33	25
	F	58	PT50	66	64	65	76	78	74
	F	64	PT52	24	67	64	62	67	71
	M	61	PT54	58	62	65	70	64	34
	F	63	PT62	57	77	62	29	60	51
	F	60	PT64	1	50	74	61	61	58
	F	60	PT65	59	52	79	77	79	82
	M	58	PT75	71	72	68	69	71	72

Table S8. SOT Condition 1 (Strategy Analysis)

Group	Sex	Age	Participant number	Pre-bed rest			Post-bed rest		
				Test 1	Test 2	Test 3	Test 1	Test 2	Test 3
Control	F	56	PT04	92	93	96	95	94	95
	M	61	PT26	95	95	95	91	94	95
	F	55	PT22	92	93	96	95	94	95
	M	66	PT27	94	94	93	95	94	93
	M	64	PT38	95	95	95	94	95	95
	M	59	PT43	93	86	94	91	93	93
	F	57	PT56	95	95	96	91	94	94
	F	57	PT68	95	94	90	94	93	93
	M	56	PT69	95	95	95	95	92	93
	M	59	PT73	93	95	95	91	94	95
	F	58	PT79	93	94	93	95	94	93
Experimental	M	57	PT21	91	94	95	91	91	95
	M	57	PT36	91	93	95	96	93	93
	M	57	PT39	91	93	92	95	94	95
	F	59	PT49	94	94	95	93	91	92
	F	58	PT50	95	95	95	96	95	95
	F	64	PT52	95	95	94	94	93	95
	M	61	PT54	16	93	94	88	95	92
	F	63	PT62	94	94	95	93	93	95
	F	60	PT64	94	95	94	94	95	94
	F	60	PT65	95	93	94	94	94	94
	M	58	PT75	96	95	95	93	92	91

Table S9. SOT Condition 2 (Strategy Analysis)

Group	Sex	Age	Participant number	Pre-bed rest			Post-bed rest		
				Test 1	Test 2	Test 3	Test 1	Test 2	Test 3
Control	F	56	PT04	94	94	94	95	94	94
	M	61	PT26	91	91	93	86	87	90
	F	55	PT22	94	94	94	95	93	86
	M	66	PT27	92	95	94	89	95	95
	M	64	PT38	95	93	95	91	91	91
	M	59	PT43	94	91	93	89	91	86
	F	57	PT56	93	96	95	91	90	95
	F	57	PT68	90	87	91	90	87	87
	M	56	PT69	95	95	94	91	94	91
	M	59	PT73	92	95	94	95	94	92
	F	58	PT79	93	95	95	95	95	94
Experimental	M	57	PT21	92	94	93	89	91	91
	M	57	PT36	91	91	93	94	91	93
	M	57	PT39	92	90	89	91	93	95
	F	59	PT49	95	93	91	92	93	91
	F	58	PT50	91	95	94	91	91	94
	F	64	PT52	91	95	94	90	93	95
	M	61	PT54	90	92	93	90	91	92
	F	63	PT62	93	93	95	93	91	91
	F	60	PT64	95	95	95	83	95	95
	F	60	PT65	91	92	93	94	91	92
	M	58	PT75	95	95	94	91	90	88

Table S10. SOT Condition 3 (Strategy Analysis)

Group	Sex	Age	Participant number	Pre-bed rest			Post-bed rest		
				Test 1	Test 2	Test 3	Test 1	Test 2	Test 3
Control	F	56	PT04	94	95	95	93	94	94
	M	61	PT26	93	93	93	79	85	89
	F	55	PT22	94	95	95	93	95	95
	M	66	PT27	66	94	94	76	88	91
	M	64	PT38	95	95	95	77	79	84
	M	59	PT43	89	95	94	91	87	92
	F	57	PT56	91	95	95	92	93	93
	F	57	PT68	72	92	91	84	74	69
	M	56	PT69	93	93	91	91	94	95
	M	59	PT73	93	93	94	92	95	94
	F	58	PT79	78	95	95	82	88	95
Experimental	M	57	PT21	86	91	94	87	93	92
	M	57	PT36	86	92	93	91	94	91
	M	57	PT39	90	93	95	89	94	94
	F	59	PT49	92	95	92	94	93	91
	F	58	PT50	92	95	95	92	93	94
	F	64	PT52	91	88	95	84	93	94
	M	61	PT54	73	93	95	82	91	92
	F	63	PT62	95	94	91	93	94	80
	F	60	PT64	93	93	95	92	95	95
	F	60	PT65	84	92	93	95	94	93
	M	58	PT75	92	95	95	91	94	93

Table S11. SOT Condition 4 (Strategy Analysis)

Group	Sex	Age	Participant number	Pre-bed rest			Post-bed rest		
				Test 1	Test 2	Test 3	Test 1	Test 2	Test 3
Control	F	56	PT04	88	90	93	82	91	88
	M	61	PT26	84	86	91	80	81	88
	F	55	PT22	88	90	93	91	86	89
	M	66	PT27	88	91	91	63	81	87
	M	64	PT38	76	90	92	73	80	80
	M	59	PT43	55	89	87	80	87	91
	F	57	PT56	91	89	92	90	87	90
	F	57	PT68	90	94	87	84	88	84
	M	56	PT69	66	90	91	71	85	87
	M	59	PT73	82	87	89	91	90	92
	F	58	PT79	91	91	92	87	91	90
Experimental	M	57	PT21	80	91	83	88	88	84
	M	57	PT36	73	86	86	77	87	88
	M	57	PT39	87	89	86	83	80	88
	F	59	PT49	76	90	88	76	90	89
	F	58	PT50	77	83	93	92	91	86
	F	64	PT52	92	91	91	90	91	91
	M	61	PT54	88	87	90	61	80	83
	F	63	PT62	86	92	92	66	90	85
	F	60	PT64	90	91	91	90	90	92
	F	60	PT65	91	89	89	92	93	91
	M	58	PT75	85	91	91	91	88	91

Table S12. SOT Condition 5 (Strategy Analysis)

Group	Sex	Age	Participant number	Pre-bed rest			Post-bed rest		
				Test 1	Test 2	Test 3	Test 1	Test 2	Test 3
Control	F	56	PT04	84	82	83	45	74	73
	M	61	PT26	48	73	74	0	41	50
	F	55	PT22	84	82	83	77	89	84
	M	66	PT27	82	75	80	64	34	67
	M	64	PT38	57	68	62	11	33	36
	M	59	PT43	79	45	68	36	66	78
	F	57	PT56	73	80	73	70	80	87
	F	57	PT68	83	87	76	62	77	66
	M	56	PT69	60	62	55	65	53	46
	M	59	PT73	67	71	76	45	68	57
	F	58	PT79	78	81	54	74	84	77
Experimental	M	57	PT21	80	79	73	78	79	81
	M	57	PT36	0	59	74	70	80	73
	M	57	PT39	66	38	78	28	51	52
	F	59	PT49	80	80	88	84	80	85
	F	58	PT50	51	78	85	77	73	84
	F	64	PT52	80	87	76	84	75	88
	M	61	PT54	51	38	60	68	76	66
	F	63	PT62	72	62	86	50	32	58
	F	60	PT64	87	85	72	76	77	84
	F	60	PT65	77	79	88	79	89	81
	M	58	PT75	80	76	86	73	79	69

Table S13. SOT Condition 6 (Strategy Analysis)

Group	Sex	Age	Participant number	Pre-bed rest			Post-bed rest		
				Test 1	Test 2	Test 3	Test 1	Test 2	Test 3
Control	F	56	PT04	84	84	85	80	80	58
	M	61	PT26	4	56	75	73	75	73
	F	55	PT22	84	84	85	82	75	80
	M	66	PT27	82	82	86	36	45	51
	M	64	PT38	21	60	74	57	64	55
	M	59	PT43	56	72	71	62	80	70
	F	57	PT56	78	72	85	78	85	90
	F	57	PT68	74	73	81	56	72	66
	M	56	PT69	54	48	71	69	84	86
	M	59	PT73	0	78	75	0	39	41
	F	58	PT79	73	87	85	76	81	84
Experimental	M	57	PT21	63	84	88	77	74	84
	M	57	PT36	73	79	59	60	76	74
	M	57	PT39	74	74	82	66	55	75
	F	59	PT49	80	84	85	82	84	78
	F	58	PT50	76	80	81	88	91	88
	F	64	PT52	72	84	89	86	87	87
	M	61	PT54	63	60	77	66	72	30
	F	63	PT62	84	88	84	63	64	38
	F	60	PT64	0	63	80	82	77	81
	F	60	PT65	82	73	81	84	87	89
	M	58	PT75	83	87	88	81	80	82

Table S14. Equilibrium Ratio – Fixed Surface

Group	Sex	Age	Participant number	Pre-bed rest	Post-bed rest
Control	F	56	PT04	0.97	0.91
	M	61	PT26	0.97	0.93
	F	55	PT22	0.97	0.98
	M	66	PT27	0.93	0.94
	M	64	PT38	1.01	0.89
	M	59	PT43	0.97	0.99
	F	57	PT56	0.98	0.98
	F	57	PT68	1.01	n/a
	M	56	PT69	0.99	0.95
	M	59	PT73	0.96	1.01
	F	58	PT79	0.97	0.91
Experimental	M	57	PT21	0.96	0.98
	M	57	PT36	0.96	0.97
	M	57	PT39	1.02	0.97
	F	59	PT49	0.96	0.76
	F	58	PT50	0.98	0.99
	F	64	PT52	0.95	0.89
	M	61	PT54	0.98	0.91
	F	63	PT62	0.98	0.95
	F	60	PT64	0.95	0.99
	F	60	PT65	1.01	0.97
	M	58	PT75	1	0.96

n/a = dropout or unable to complete

Table S15. Equilibrium Ratio – Sway/Ref Surface

Group	Sex	Age	Participant number	Pre-bed rest	Post-bed rest
Control	F	56	PT04	1.19	0.75
	M	61	PT26	0.8	0.97
	F	55	PT22	0.8	0.96
	M	66	PT27	0.5	0.86
	M	64	PT38	0.87	1.03
	M	59	PT43	0.67	0.83
	F	57	PT56	0.61	0.75
	F	57	PT68	0.59	n/a
	M	56	PT69	0.78	0.79
	M	59	PT73	0.83	0.99
	F	58	PT79	0.71	0.66
Experimental	M	57	PT21	0.66	0.7
	M	57	PT36	0.92	0.87
	M	57	PT39	1.57	0.83
	F	59	PT49	0.77	1.14
	F	58	PT50	0.76	0.84
	F	64	PT52	0.95	0.45
	M	61	PT54	0.66	0.71
	F	63	PT62	0.95	0.99
	F	60	PT64	0.8	0.89
	F	60	PT65	1.17	0.78
	M	58	PT75	1.04	0.91

n/a = dropout or unable to complete

Table S16. Equilibrium Score for SOT-2M Condition

Group	Sex	Age	Participant number	Pre-bed rest						Post-bed rest					
				Test 1	Test 2	Test 3	Test 4	Test 5	Test 6	Test 1	Test 2	Test 3	Test 4	Test 5	Test 6
Control	F	56	PT04	87	91	91	89	94	89	92	84	88	84	84	89
	M	61	PT26	94	93	88	94	93	92	89	97	91	76	85	95
	F	55	PT22	90	84	85	88	86	90	75	82	85	89	92	87
	M	66	PT27	83	82	83	83	84	91	87	87	75	90	81	79
	M	64	PT38	70	93	90	87	90	86	55	72	73	87	86	76
	M	59	PT43	87	85	91	88	89	89	87	88	89	85	93	91
	F	57	PT56	93	93	93	91	88	93	92	91	91	92	89	94
	F	57	PT68	88	83	82	90	81	88	n/a					
	M	56	PT69	91	93	91	92	86	78	89	78	86	86	85	85
	M	59	PT73	84	90	91	89	88	85	95	93	94	93	94	94
	F	58	PT79	81	86	92	88	90	92	89	93	81	86	83	86
Experimental	M	57	PT21	87	90	87	86	89	90	87	90	59	92	90	86
	M	57	PT36	86	90	89	85	78	82	85	79	87	89	83	78
	M	57	PT39	90	92	94	93	94	91	87	91	90	92	92	90
	F	59	PT49	86	87	86	84	88	90	92	48	70	76	77	65
	F	58	PT50	89	89	91	91	87	85	92	86	95	89	90	76
	F	64	PT52	92	90	90	90	84	84	89	83	67	82	86	84
	M	61	PT54	88	88	83	90	86	87	91	67	85	89	84	85
	F	63	PT62	88	87	87	91	89	88	82	87	83	89	89	87
	F	60	PT64	92	88	87	90	92	91	93	91	84	90	92	90
	F	60	PT65	94	91	90	94	94	95	88	92	90	93	83	89
	M	58	PT75	90	92	92	92	95	94	85	92	91	88	90	84

n/a = dropout or unable to complete

Table S17. Vertical Velocity (degree/second) for SOT-M2 Condition

Group	Sex	Age	Participant number	Pre-bed rest						Post-bed rest					
				Test 1	Test 2	Test 3	Test 4	Test 5	Test 6	Test 1	Test 2	Test 3	Test 4	Test 5	Test 6
Control	F	56	PT04	40	53	38	34	38	34	48	51	53	53	51	49
	M	61	PT26	40	37	38	36	30	32	22	21	20	21	24	23
	F	55	PT22	35	32	34	30	34	29	31	34	31	33	31	28
	M	66	PT27	39	39	34	31	33	32	33	29	30	33	31	30
	M	64	PT38	24	23	23	24	23	25	22	20	20	17	17	17
	M	59	PT43	41	44	35	36	25	31	66	74	37	33	28	32
	F	57	PT56	39	45	42	43	42	38	27	32	29	32	32	33
	F	57	PT68	37	39	40	42	41	38	n/a					
	M	56	PT69	47	44	45	49	48	44	37	35	35	36	36	34
	M	59	PT73	57	59	54	59	55	60	28	31	32	38	32	32
	F	58	PT79	56	44	42	41	41	42	45	44	46	46	46	44
Experimental	M	57	PT21	54	48	54	59	54	56	45	40	43	43	41	42
	M	57	PT36	44	34	32	26	28	33	41	45	53	51	54	52
	M	57	PT39	40	41	42	44	45	43	41	47	44	42	41	39
	F	59	PT49	53	72	65	55	50	54	37	31	32	30	32	36
	F	58	PT50	47	47	46	49	50	38	39	39	38	37	36	36
	F	64	PT52	31	31	35	39	36	33	48	52	50	56	60	58
	M	61	PT54	34	43	49	50	50	50	44	43	43	50	50	50
	F	63	PT62	46	37	28	29	32	28	43	29	31	31	34	37
	F	60	PT64	36	39	46	45	48	49	27	32	32	34	34	34
	F	60	PT65	28	27	35	36	30	32	18	19	18	18	17	18
	M	58	PT75	25	26	29	27	28	27	24	26	24	27	24	24

n/a = dropout or unable to complete

Table S18. Equilibrium Score for SOT-M5 Condition

Group	Sex	Age	Participant number	Pre-bed rest						Post-bed rest					
				Test 1	Test 2	Test 3	Test 4	Test 5	Test 6	Test 1	Test 2	Test 3	Test 4	Test 5	Test 6
Control	F	56	PT04	79	61	80	77	82	50	49	31	45	41	46	31
	M	61	PT26	19	50	53	56	56	52	51	56	56	57	54	54
	F	55	PT22	21	69	36	76	47	33	27	56	68	45	56	52
	M	66	PT27	23	23	17	25	41	40	11	53	48	45	30	13
	M	64	PT38	49	61	53	46	38	66	51	51	53	67	59	52
	M	59	PT43	27	51	55	51	31	32	48	52	68	62	57	39
	F	57	PT56	27	47	35	24	11	57	30	58	52	42	31	57
	F	57	PT68	41	26	45	29	40	10	n/a					
	M	56	PT69	39	47	50	72	62	58	66	45	56	58	64	68
	M	59	PT73	33	63	46	56	27	62	55	71	61	84	70	57
	F	58	PT79	22	34	49	30	36	45	24	38	51	12	19	39
Experimental	M	57	PT21	1	19	28	28	49	47	47	79	65	25	58	27
	M	57	PT36	45	53	71	61	55	64	39	55	44	56	51	70
	M	57	PT39	66	82	83	79	80	91	11	54	45	51	62	67
	F	59	PT49	29	54	23	44	37	43	39	5	53	36	56	44
	F	58	PT50	51	43	52	52	23	34	31	61	45	69	55	48
	F	64	PT52	17	56	53	64	54	48	22	32	36	35	5	51
	M	61	PT54	23	47	37	38	40	33	58	29	50	54	33	52
	F	63	PT62	55	58	23	38	43	52	9	44	55	72	60	57
	F	60	PT64	32	27	46	32	52	55	55	47	47	50	23	65
	F	60	PT65	39	62	74	68	82	59	44	54	42	50	49	57
	M	58	PT75	59	73	73	55	80	84	51	49	86	75	55	64

n/a = dropout or unable to complete

Table S19. Vertical Velocity (degree/second) for SOT-M5 Condition

Group	Sex	Age	Participant number	Pre-bed rest						Post-bed rest					
				Test 1	Test 2	Test 3	Test 4	Test 5	Test 6	Test 1	Test 2	Test 3	Test 4	Test 5	Test 6
Control	F	56	PT04	37	37	37	34	35	37	58	55	57	52	54	58
	M	61	PT26	42	40	35	38	36	35	24	24	26	25	24	30
	F	55	PT22	32	32	30	30	34	30	30	27	31	26	32	30
	M	66	PT27	34	33	30	34	34	35	24	30	27	31	30	29
	M	64	PT38	26	27	29	30	34	31	16	17	15	13	13	14
	M	59	PT43	45	41	46	49	44	46	43	43	38	39	41	41
	F	57	PT56	46	49	52	45	50	44	43	38	44	45	45	48
	F	57	PT68	48	45	47	53	53	55	n/a					
	M	56	PT69	42	43	41	36	31	36	35	32	34	37	37	34
	M	59	PT73	40	42	44	42	44	40	37	40	38	37	42	42
	F	58	PT79	41	41	44	44	47	48	55	54	54	51	48	48
Experimental	M	57	PT21	65	75	70	72	69	67	52	47	49	58	50	51
	M	57	PT36	31	35	37	34	35	35	54	59	45	45	48	50
	M	57	PT39	42	37	37	36	32	40	43	37	36	35	36	34
	F	59	PT49	55	50	57	54	50	45	37	33	37	35	31	26
	F	58	PT50	30	27	29	21	27	26	35	32	32	30	35	35
	F	64	PT52	44	47	44	43	42	39	62	56	53	50	56	59
	M	61	PT54	58	49	51	57	56	55	44	52	48	53	54	39
	F	63	PT62	31	22	21	23	17	18	27	31	23	22	18	21
	F	60	PT64	60	72	58	63	67	58	39	40	43	38	36	40
	F	60	PT65	37	38	31	33	32	34	20	21	21	22	21	22
	M	58	PT75	27	26	27	33	30	23	30	28	24	27	27	27

n/a = dropout or unable to complete

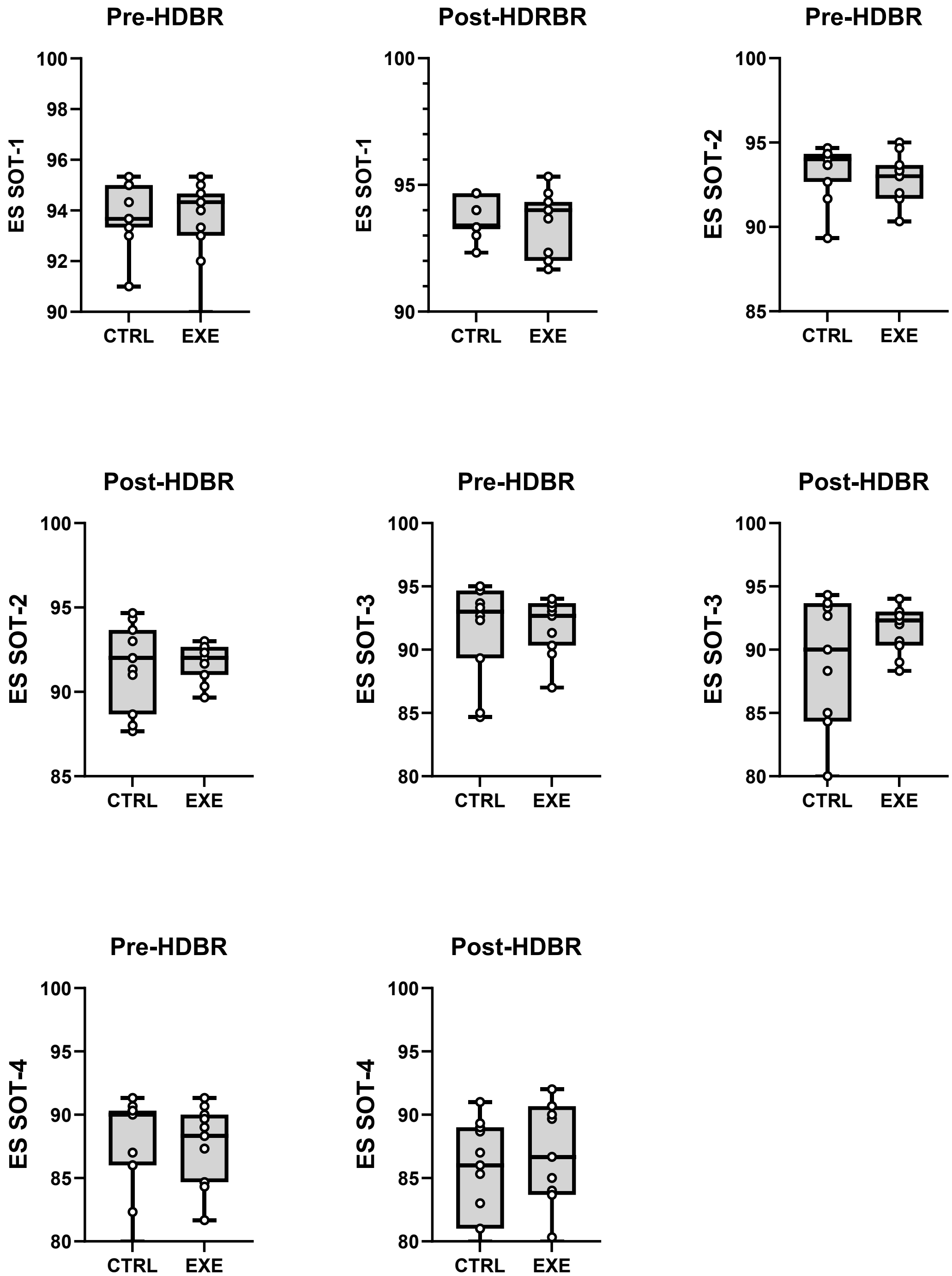

Figure S1. Box-and-whisker plots with overlaid individual data points showing equilibrium score (ES) before (Pre) and after (Post) head-down bed rest (HDBR) for Sensory Organization Test (SOT) conditions 1–4. Data are presented separately for the exercise (EXE, n = 11) and control (CTRL, n = 11) groups. Boxes represent the interquartile range (IQR) with the median indicated, whiskers denote the full range, and scatter points correspond to individual participants.

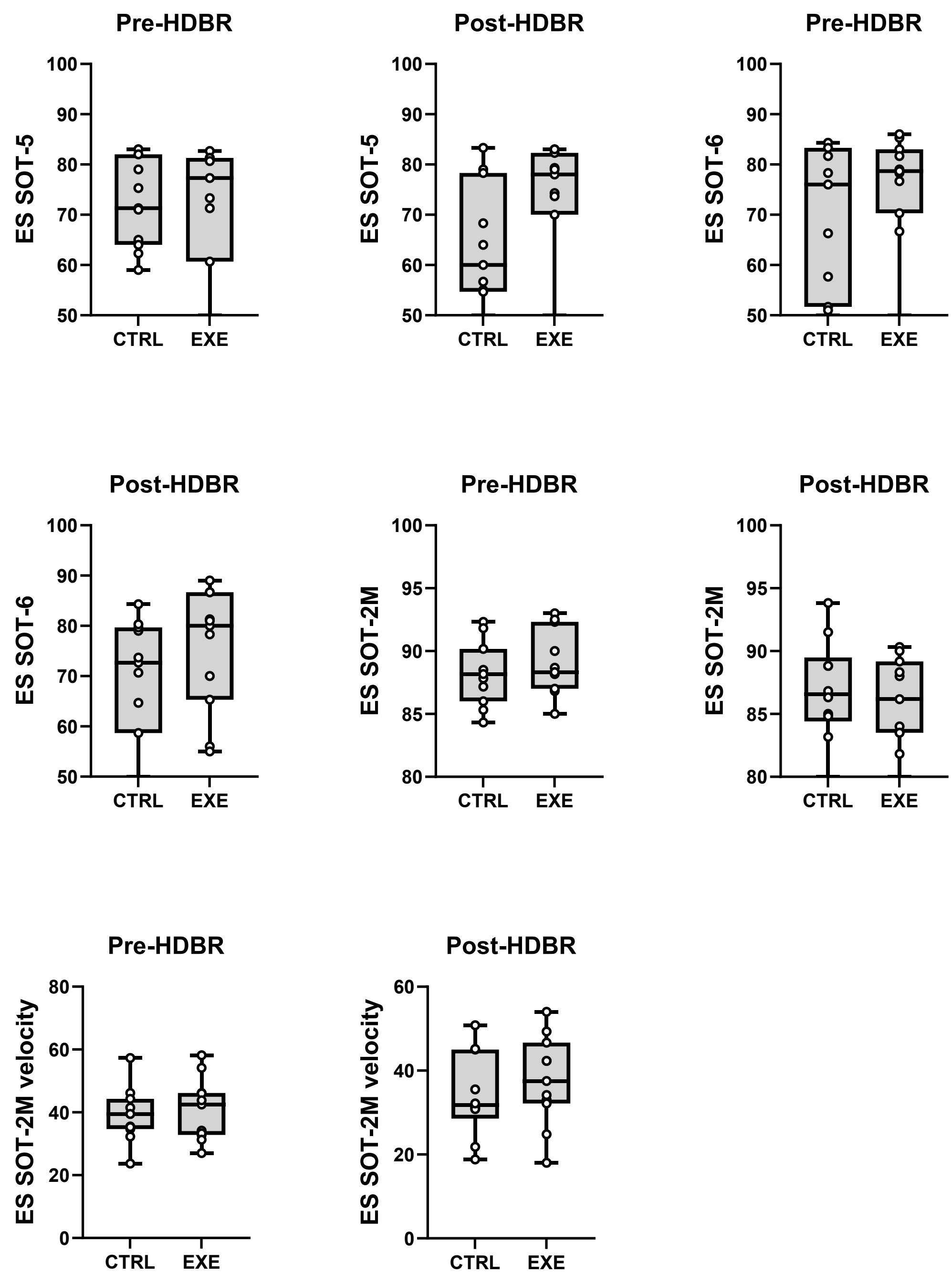

Figure S2. Box-and-whisker plots with overlaid individual data points showing pre- (Pre) and post- (Post) head-down bed rest (HDBR) performance for Sensory Organization Test (SOT) conditions 5–6 and the head-shake condition SOT-2M. Outcomes include equilibrium score (ES) for SOT conditions 5–6 and SOT-2M, and vertical velocity (vv) for SOT-2M. Data are presented for the exercise (EXE, n = 11) and control (CTRL, n = 11) groups. Boxes represent the interquartile

range (IQR) with the median indicated, whiskers denote the full range, and scatter points correspond to individual participants.

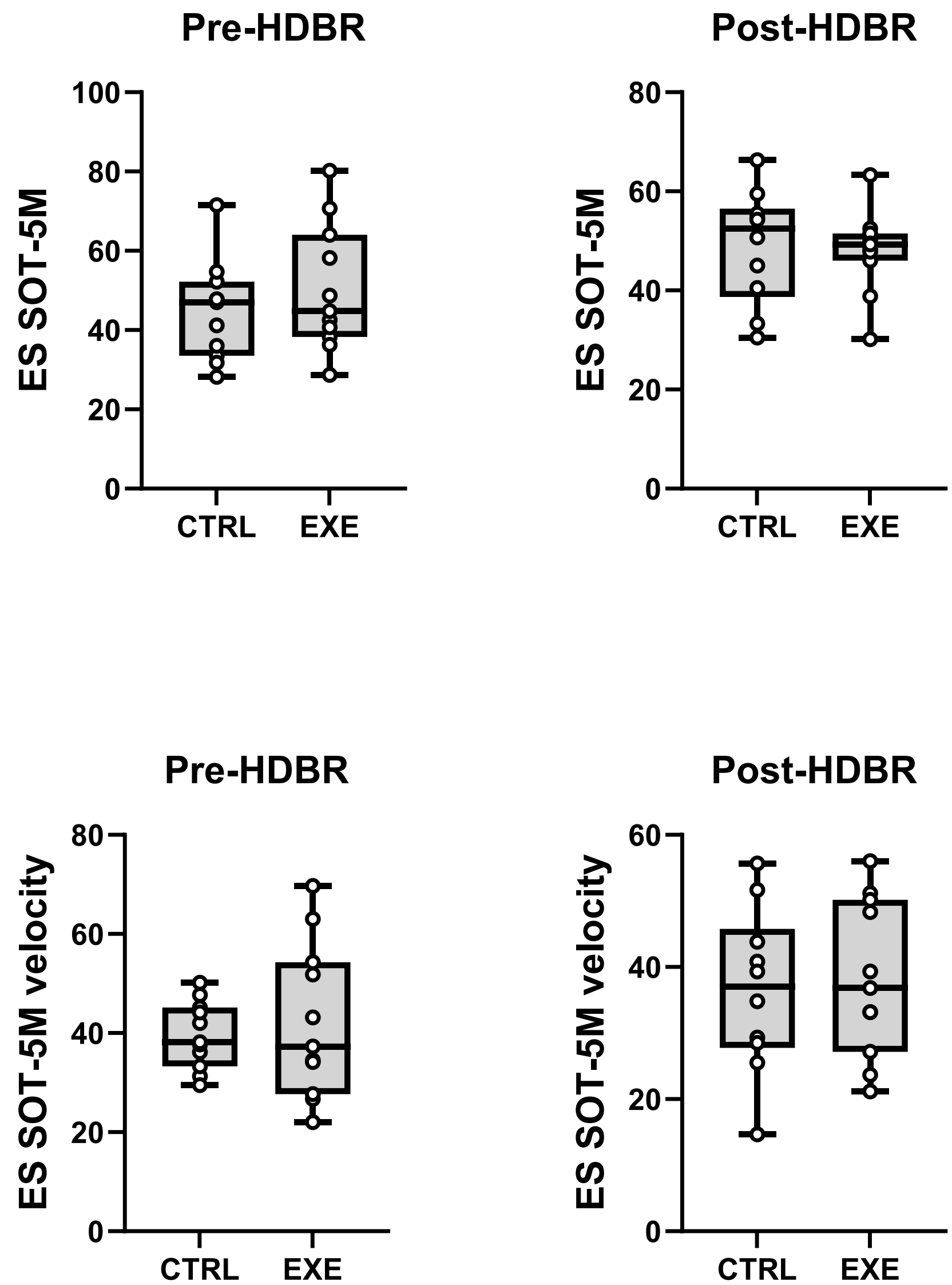

Figure S3. Box-and-whisker plots with overlaid individual data points showing pre- (Pre) and post- (Post) head-down bed rest (HDBR) performance for the head-shake condition SOT-5M. Four panels present the assessed outcomes (e.g., equilibrium score [ES] and vertical velocity [vv]) for the exercise (EXE, n = 11) and control (CTRL, n = 11) groups. Boxes represent the interquartile range (IQR) with the median indicated, whiskers denote the full range, and scatter points correspond to individual participants.

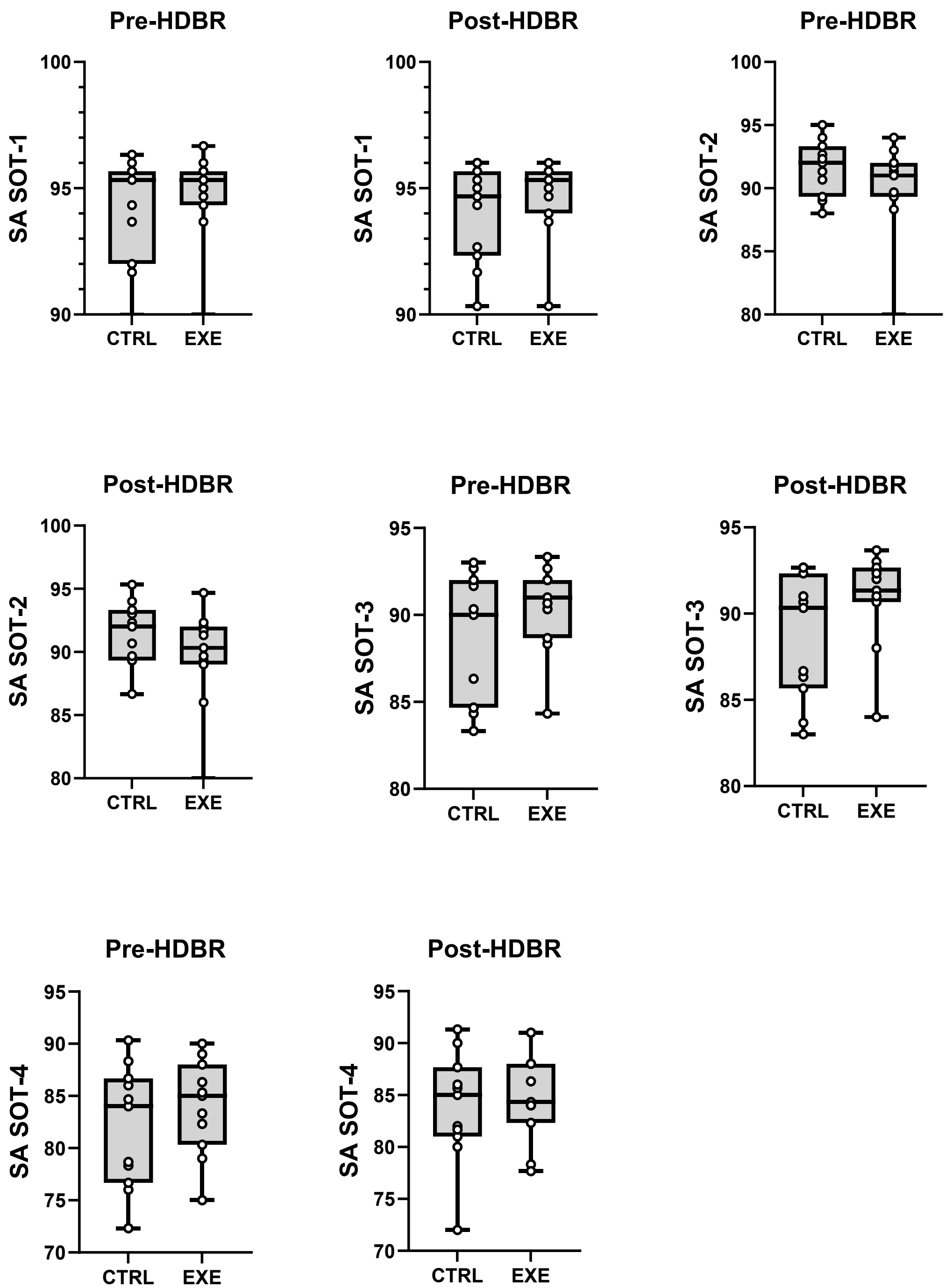

Figure S4. Box-and-whisker plots with overlaid individual data points showing strategy analysis score (SA) before (Pre) and after (Post) head-down bed rest (HDBR) for Sensory Organization Test (SOT) conditions 1–4. Data are presented separately for the exercise (EXE, n = 11) and control (CTRL, n = 11) groups. Boxes represent the interquartile range (IQR) with the median indicated, whiskers denote the full range, and scatter points correspond to individual participants.

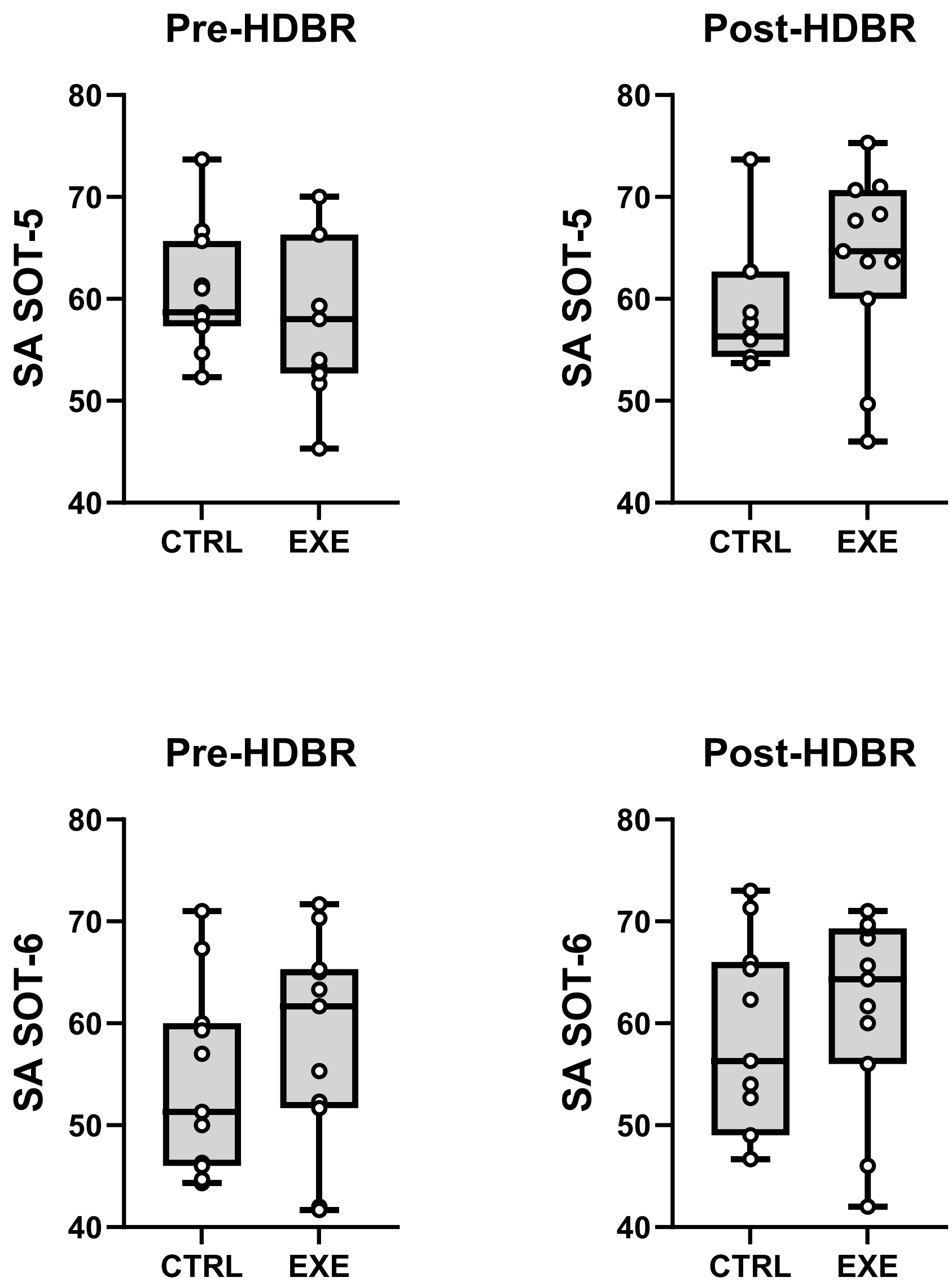

Figure S5. Box-and-whisker plots with overlaid individual data points showing pre- (Pre) and post- (Post) head-down bed rest (HDBR) performance for Sensory Organization Test (SOT) conditions 5–6 and the head-shake condition SOT-2M. Outcomes include strategy analysis score (SA) for SOT conditions 5–6 and SOT-2M, and vertical velocity (vv) for SOT-2M. Data are presented for the exercise (EXE, n = 11) and control (CTRL, n = 11) groups. Boxes represent the interquartile range (IQR) with the median indicated, whiskers denote the full range, and scatter points correspond to individual participants.

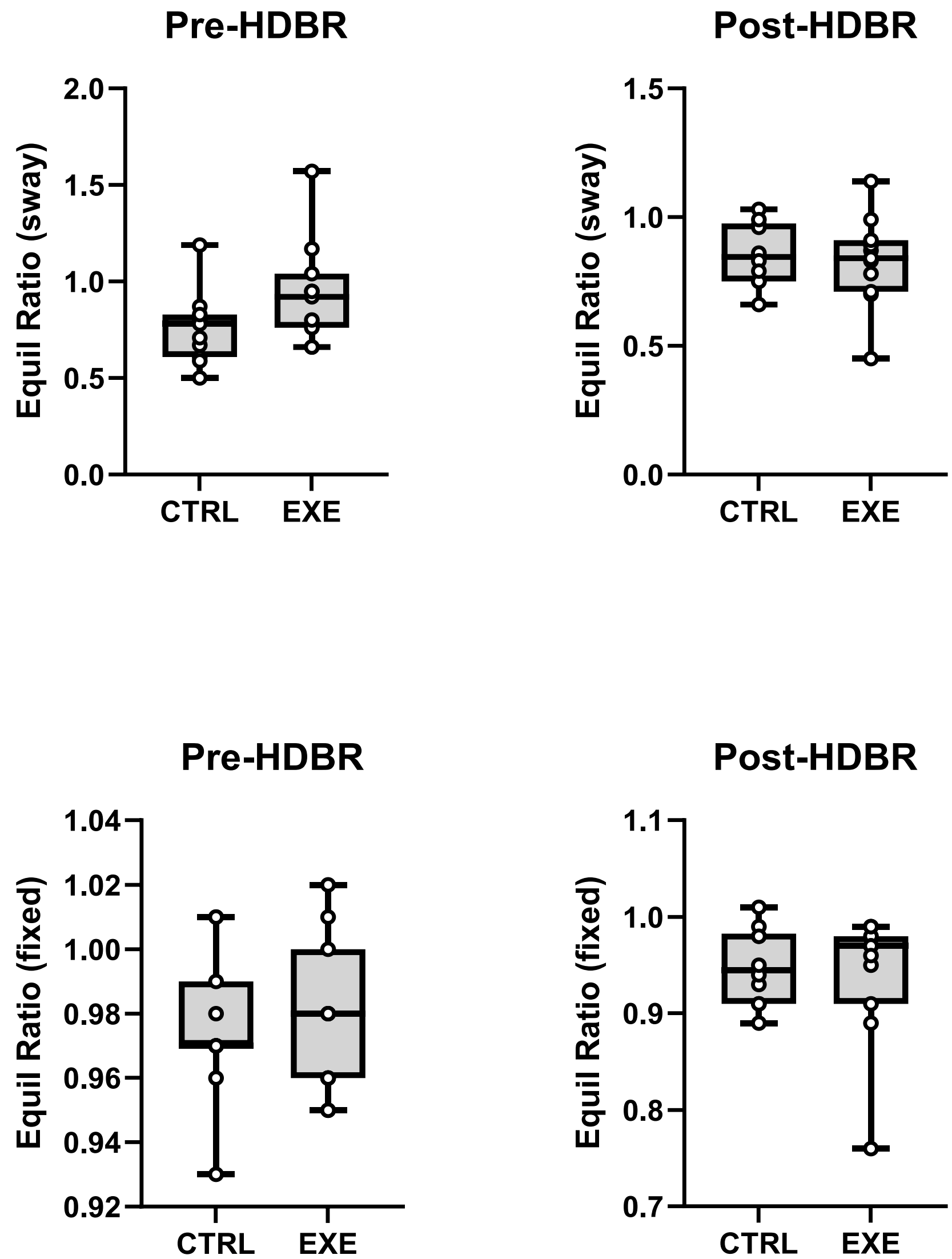

Figure S6. Box-and-whisker plots with overlaid individual data points showing pre- (Pre) and post- (Post) head-down bed rest (HDBR) performance for equilibrium ratio, fixed, and sway measures in the exercise (EXE, n = 11) and control (CTRL, n = 11) groups. Boxes represent the interquartile range (IQR) with the median indicated, whiskers denote the full range, and scatter points correspond to individual participants.

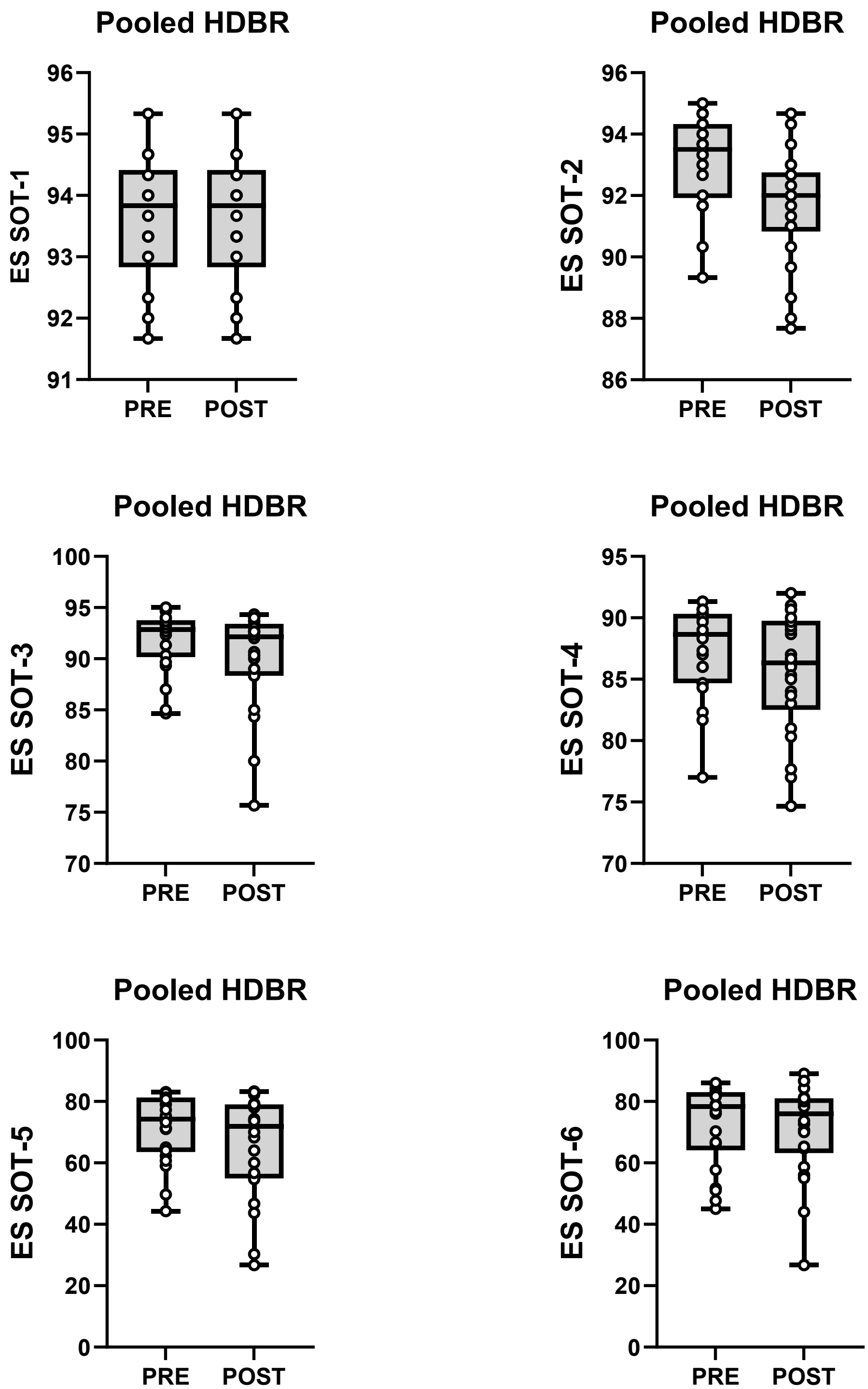

Figure S7. Box-and-whisker plots with overlaid individual data points showing equilibrium score (ES) before (Pre) and after (Post) head-down bed rest (HDBR) for pooled Sensory Organization Test (SOT) conditions 1–6. Data are presented separately for the exercise (EXE, n = 11) and control (CTRL, n = 11) groups. Boxes represent the interquartile range (IQR) with the median indicated, whiskers denote the full range, and scatter points correspond to individual participants.

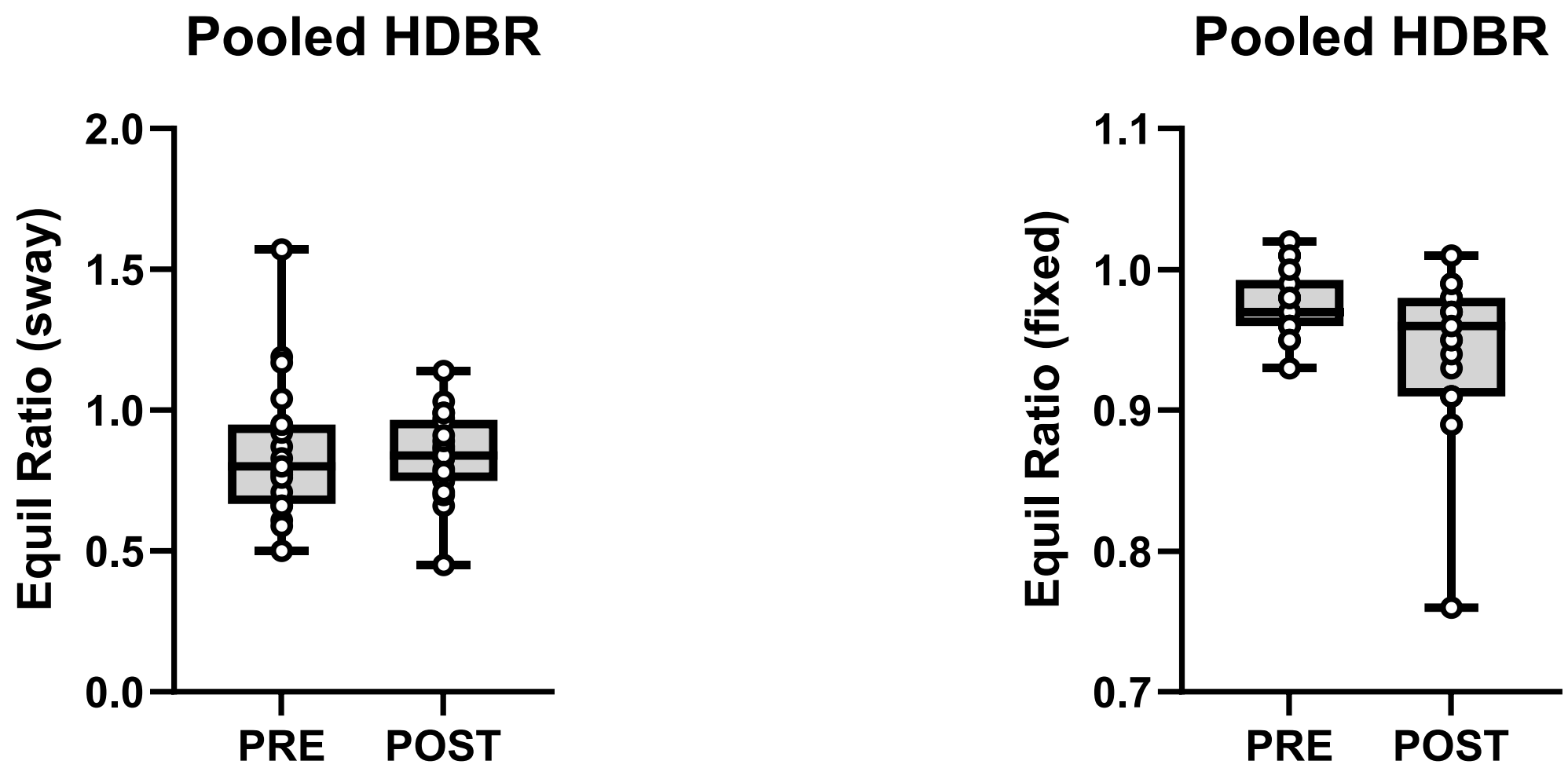

Figure S8. Box-and-whisker plots with overlaid individual data points showing pre- (Pre) and post- (Post) head-down bed rest (HDBR) performance for pooled equilibrium ratio, fixed, and sway measures (across conditions) in the exercise (EXE, n = 11) and control (CTRL, n = 11) groups. Boxes represent the interquartile range (IQR) with the median indicated, whiskers denote the full range, and scatter points correspond to individual participants.
